# FlowMax: A Computational Tool for Maximum Likelihood Deconvolution of CFSE Time Courses

**DOI:** 10.1371/journal.pone.0067620

**Published:** 2013-06-27

**Authors:** Maxim Nikolaievich Shokhirev, Alexander Hoffmann

**Affiliations:** 1 Signaling Systems Laboratory, Department of Chemistry and Biochemistry, University of California San Diego, La Jolla, California, United States of America; 2 San Diego Center for Systems Biology, La Jolla, California, United States of America; 3 Graduate Program in Bioinformatics and Systems Biology, University of California San Diego, La Jolla, California, United States of America; University of Leeds, United Kingdom

## Abstract

The immune response is a concerted dynamic multi-cellular process. Upon infection, the dynamics of lymphocyte populations are an aggregate of molecular processes that determine the activation, division, and longevity of individual cells. The timing of these single-cell processes is remarkably widely distributed with some cells undergoing their third division while others undergo their first. High cell-to-cell variability and technical noise pose challenges for interpreting popular dye-dilution experiments objectively. It remains an unresolved challenge to avoid under- or over-interpretation of such data when phenotyping gene-targeted mouse models or patient samples. Here we develop and characterize a computational methodology to parameterize a cell population model in the context of noisy dye-dilution data. To enable objective interpretation of model fits, our method estimates fit sensitivity and redundancy by stochastically sampling the solution landscape, calculating parameter sensitivities, and clustering to determine the maximum-likelihood solution ranges. Our methodology accounts for both technical and biological variability by using a cell fluorescence model as an adaptor during population model fitting, resulting in improved fit accuracy without the need for *ad hoc* objective functions. We have incorporated our methodology into an integrated phenotyping tool, FlowMax, and used it to analyze B cells from two NFκB knockout mice with distinct phenotypes; we not only confirm previously published findings at a fraction of the expended effort and cost, but reveal a novel phenotype of nfkb1/p105/50 in limiting the proliferative capacity of B cells following B-cell receptor stimulation. In addition to complementing experimental work, FlowMax is suitable for high throughput analysis of dye dilution studies within clinical and pharmacological screens with objective and quantitative conclusions.

## Introduction

Lymphocyte population dynamics within the mammalian immune response have been extensively studied, as they are a predictor of vaccine efficacy, while their misregulation may lead to cancers or autoimmunity [1]. Lymphocyte population dynamics involve seemingly stochastic cellular parameters describing the decision to respond to the stimulus, the time spent progressing through the cell cycle, the time until programmed cell death, and the number of divisions progenitor cells undergo [2]. Specifically, experimental observations show that population dynamics are well modeled at the cellular level by skewed distributions for the time to divide and die, that these distributions are different for undivided and dividing cells, and that the proliferative capacity is limited [3]. Recently, Hawkins *et al* showed that cells, that exhibit growth in size invariably divide (though at highly variable times), while cells that do not are committed to cell death, albeit at highly variable times [3]. A high degree of biological variability may ensure that population-level immune responses are robust [2], [4], but renders the deconvolution of experimental data and their subsequent interpretation challenging.

A current experimental approach for tracking lymphocyte population dynamics involves flow cytometry of carboxyfluorescein succimidyl ester (CFSE)-stained cells. First introduced in 1990 [5], CFSE tracking relies on the fact that CFSE is irreversibly bound to proteins in cells, resulting in progressive halving of cellular fluorescence with each cell division. By measuring the fluorescence of thousands of cells at various points in time after stimulation, fluorescence histograms with peaks representing generations of divided cells are obtained. However, interpreting CFSE data confronts two challenges. In addition to intrinsic biological complexity arising from generation- and cell age-dependent variability in cellular processes, fluorescence signals for a specific generation are not truly uniform due to heterogeneity in (i) staining of the founder population, (ii) partitioning of the dye during division, and (iii) dye clearance from cells over time. Thus, while high-throughput experimental approaches enable population-level measurements, deconvolution of CFSE time courses into biologically-intuitive cellular parameters is susceptible to misinterpretation [6].

To recapitulate lymphocyte population dynamics a number of theoretical models have been developed (see [7], [8] for recent reviews). However, the available computational methodologies to utilize them for analyzing CFSE time series data remain cumbersome, and these are prone to under- or over- interpretation. First, commercial software such as FlowJo (Tree Star Inc.) and FCExpress (De Novo Software) is typically used to fit Gaussian distributions to log-fluorescence data on a histogram-by-histogram basis to determine cell counts at each generation, but these do not provide an objective measure of fit quality. Then mathematical models of population dynamics must be employed to fit cell cycle and cell death parameters to the fitted generational cell counts [9], [10]; however, they also do not provide a measure of fit quality, and they are affected by errors in cell-counts determined by aforementioned software tools. Without an estimate of solution sensitivity and redundancy in the quantitative conclusions, computational tools do not give a sense of whether the information contained in CFSE data is used appropriately (or whether it is under- or over-interpreted). This may be the underlying reason for why population dynamic models have not yet impacted experimental or clinical research for the interpretation of ubiquitous CFSE data.

Here, we introduce an integrated computational methodology for phenotyping lymphocyte expansion in terms of single-cell parameters. We first evaluate the theoretical accuracy of each module in the phenotyping process by fitting generated data. We then show that implementing them in an integrated, rather than sequential, workflow reduces expected parameter error. Next, we describe our approach to estimating the quality of the fit and demonstrate the advantages of using our integrated methodology compared to phenotyping with the current state-of-the-art approach, the Cyton Calculator [9]. We then evaluate how different types of imperfections in data quality affect performance. Finally, we demonstrate the method’s utility in phenotyping B cells from *nfkb1^−/−^* and *rel^−/−^* mice stimulated with anti-IgM and LPS, extending the conclusions of previously published studies [11], [12] and disaggregating the role of distinct cellular parameters by using the model simulation capabilities. FlowMax, a Java tool implementation of our methodology as well as the experimental datasets are available for download from http://signalingsystems.ucsd.edu/models-and-code/.

## Results

**Figure 1:**
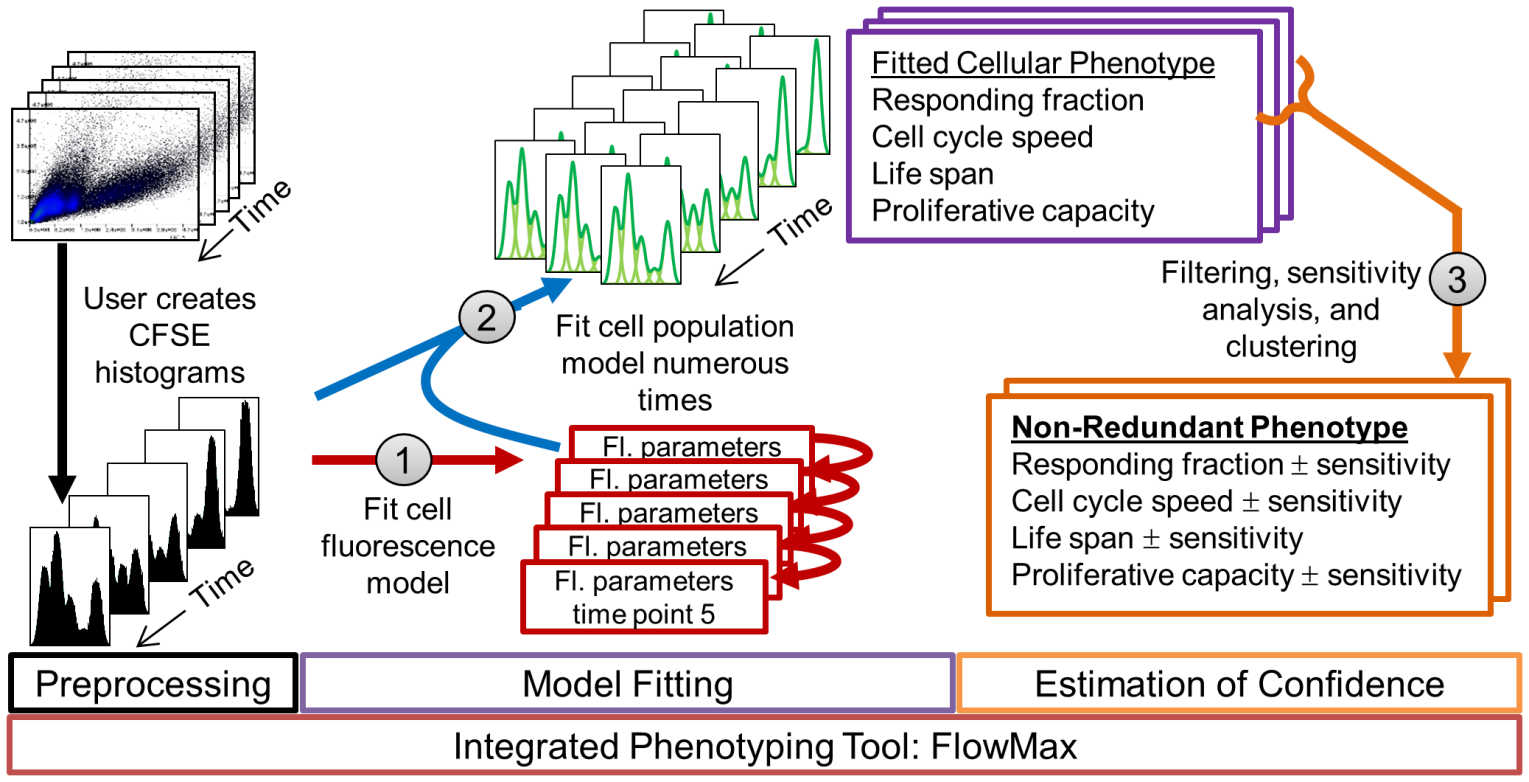
Proposed integrated phenotyping approach (FlowMax). CFSE flow-cytometry time series are preprocessed to create one-dimensional fluorescence histograms that are used to determine the cell proliferation parameters for each time point, using the parameters of the previous time points as added constraints (step 1). Fluorescence parameters are then used to extend a cell population model and allow for direct training of the cell population parameters on the fluorescence histograms (step 2). To estimate solution sensitivity and redundancy, step 2 is repeated many times, solutions are filtered by score, parameter sensitivities are determined for each solution, non-redundant maximum-likelihood parameter ranges are found after clustering, and a final filtering step eliminates clusters representing poor solutions (step 3).

To enable objective interpretation of dye dilution lymphocyte proliferation studies, we constructed a suite of integrated computational modules (Figure 1). Given a CFSE dye-dilution time course, the first step involves fitting the cell fluorescence model to CFSE fluorescence histograms recorded at various times, accounting for dye dilution from cell division and intrinsic variability from biological and technical sources. In a second step, a cell population model, describing the fraction of responding cells in each generation and times to cell division or death, is fit to the CFSE time series data directly, using the best-fit cell fluorescence parameters as adaptors during fitting. Repeating the second fitting step numerous times allows for a critical third step: estimating the sensitivity and degeneracy of the best fit parameter set, providing the maximum likelihood non-redundant solutions ranges.

### Evaluating the Accuracy of Cell Fluorescence Model Fitting

**Figure 2:**
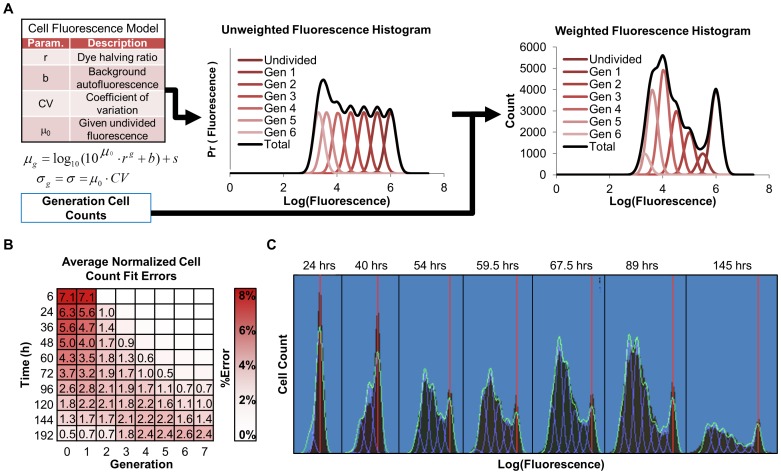
The cell fluorescence model. (A) Noisy log-transformed cell fluorescence is modeled by a weighted mixture of Gaussian distributions for each cell division: 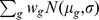, parameterized according to equations describing variability in staining (CV), background fluorescence (b), dye dilution (r), and a small correction for the fluorescence of the initial population of cells (s). Weights for each Gaussian correspond to cell counts in each generation. (B) Analysis of the cell fluorescence model fitting accuracy for 1,000 generated CFSE fluorescence time courses (see also Tables S3 and S4). Average percent error in generational cell counts normalized to the maximum generational cell count for each time course. Numbers indicate an error ≥ 0.5%. (C) Representative cell fluorescence model fitting to experimental data from wildtype B cells at indicated time points after start of lipopolysaccharides (LPS) stimulation (red lines indicate undivided population).

The first computational module addresses the challenge of converting fluorescence histograms of CFSE data into generation-specific cell counts and experimental dye parameters. We selected a simple time-independent cell fluorescence model (Figure 2A) similar to the models used in current flow cytometry analysis tools (TreeStar Inc., De Novo Software) and recent studies [13]–[15]. We assume that the log-transformed fluorescence of populations of cells is well-modeled by a mixture of Gaussians, as observed previously [9]. We selected this simple model because recent models [13], [16]–[18], which incorporate both cell dynamics and dye dynamics, do not naturally account for both cell age-dependent death and division rates, as well as for the observation that only a fraction of lymphocytes choose to respond to the stimulus. While the cell fluorescence model does not explicitly account for time-dependent dye catabolism, the model allows for the fluorescence of the initial population, 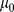, to be manually specified for each time point when log-fluorescence histograms are constructed.

In order to quantify the cell fluorescence model fitting accuracy, we tested it with a panel of generated realistic CFSE time courses. Specifically, the cell fluorescence model was fitted to the generated histograms and the average normalized % error between generated and fitted peak counts as a function of time point (Figure 2B). As expected, the average error in generation counts was highest for early time points due to absence of a second peak, which may help constrain parameter fitting. However, the % error between generated and fitted peak counts (Figure 2B) suggested that the fluorescence model fitting was on average quite successful as the maximum average normalized error was 7.1%. Finally, direct comparison of cell fluorescence model fits to experimental data showed good agreement throughout the entire time course, even when late generation peaks are poorly resolved (Figure 2C).

### Evaluating the Accuracy of Cell Population Model Fitting

**Figure 3:**
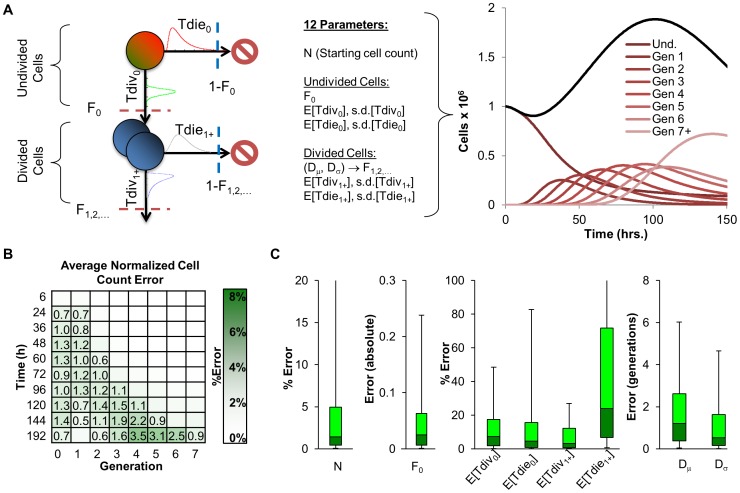
The fcyton cell proliferation model. (A) A graphical representation summarizing the model parameters required to calculate the total number of cells in each generation as a function of time. Division and death times are assumed to be log-normally distributed and different between undivided and dividing cells. Progressor fractions (Fs) determine the fraction of responding cells in each generation committed to division and protected from death. (B,C) Analysis of the accuracy associated with fitting fcyton parameters for a set of 1,000 generated realistic datasets of generational cell counts assuming perfect cell counts and an optimized *ad hoc* objective function (see Text S1 and Tables S3 and S4). (B) Average percent error in generational cell counts normalized to the maximum generational cell count for each time course. Numbers indicate an error ≥ 0.5%. (C) Analysis of the error associated with determining key fcyton parameters. Box plots represent 5, 25, 50, 75, and 95 percentile values. Outliers are not shown. For analysis of all fcyton parameter errors see also Figure S2 (green).

Employing the fcyton model described above (Figure 3A), we examined the accuracy associated with fitting the fcyton population model with the generated panel of datasets directly to the known generational cell counts, and calculated both the average normalized cell count error (Figure 3B) as well as the error distributions associated with fitting particular fcyton parameters (Figure 3C). Fitting the fcyton model to given counts resulted in very low generational cell count errors : the maximum average normalized error was 3.5%, while the maximum average normalized error for all time points ≤120 h was always less than 2%. The median errors in the key parameters N, F_0_, E[Tdiv_0_], E[Tdie_0_]_,_ E[Tdiv_1+_]) were small: 1.2%, 0.02, 5.8%,4.0%, and 2.6%, respectively. However, interestingly, even with perfect knowledge of generational cell counts and a large number of time points, not all cellular parameters were accurately determined. This is illustrated by a median % error value of about 18% for E[Tdie_1+_] and a median error of about 1 generation for Dμ, the average number of divisions a divided cell will undergo, and suggests that these parameters do not contribute substantially to the cell count data within the physiologically relevant parameter regime.

### Evaluating Accuracy when both Model Fitting Steps are Incorporated

Interpreting the population dynamics provided by dye dilution data in terms of cellular parameters requires both computational modules: the cell fluorescence model describes variability in experimental staining, while cell proliferation modeling explains evolution of the population through time. We first assessed their performance when linked sequentially, fitting the population model to best-fit cell counts, using the above-described generated dataset. Since the objective function that determines the fit of model output to experimental cell counts is a key determinant of the performance, we compared a simple squared deviation scoring function (SD) with a more complex, manually-optimized objective function which takes into account multiple measures of similarity (Equations 27 and 28 in Text S1). The results showed that a complex *ad hoc* optimized scoring function drastically outperformed the simpler SD-based scoring function with all fcyton parameter error distributions significantly (each p-value <1E-12; Mann-Whitney U test) shifted toward zero (Figure S1).

**Figure 4:**
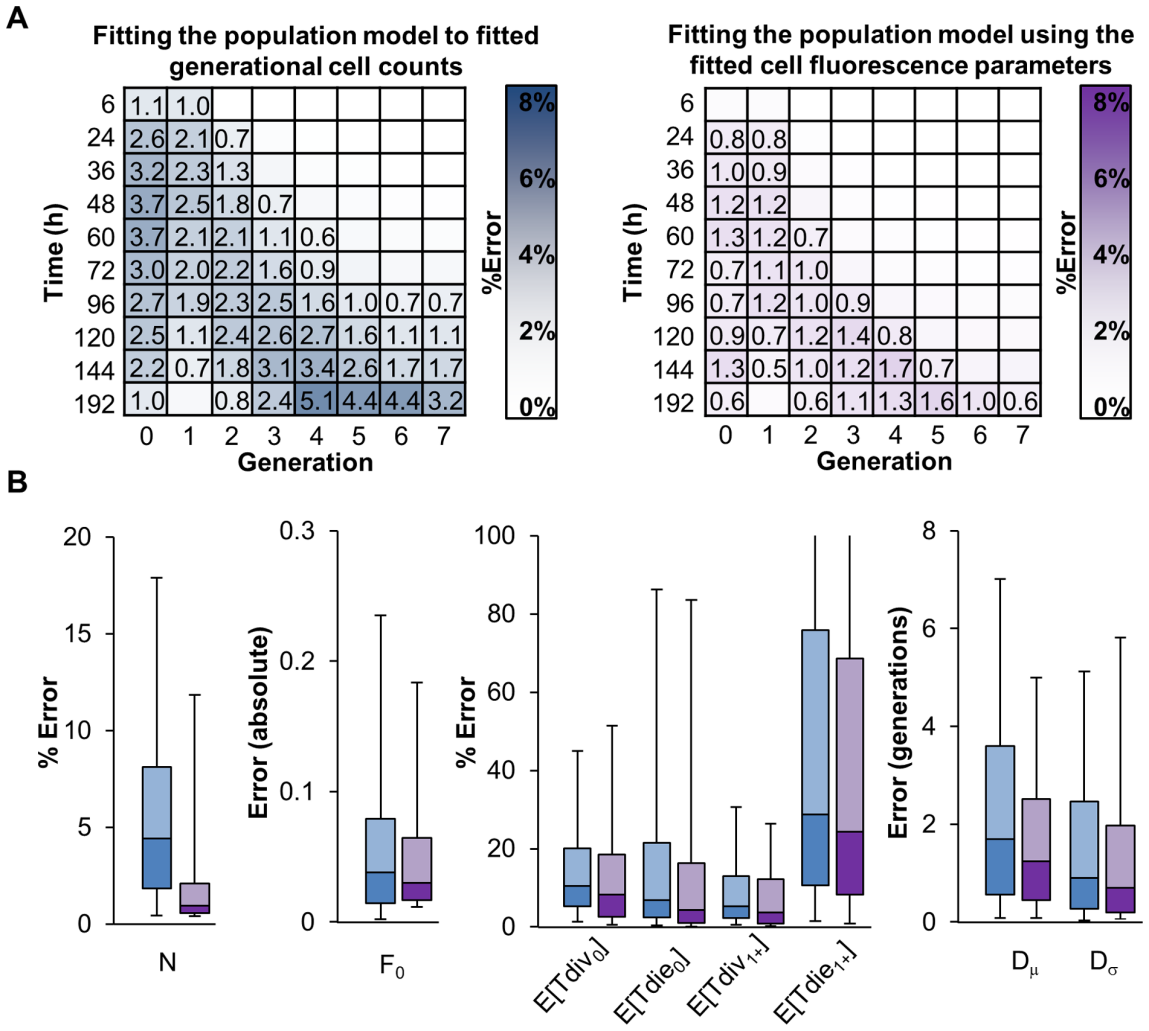
Accuracy of phenotyping generated datasets in a sequential or integrated manner. The accuracy associated with sequential fitting Gaussians to fluorescence data to obtain cell counts for each generation (blue) and integrated fitting of the fcyton model to fluorescence data directly using fitted fluorescence parameters as adaptors (purple) was determined for 1,000 sets of randomly generated realistic CFSE time courses (see also Tables S3 and S4). (A) Average percent error in generational cell counts normalized to the maximum generational cell count for each time course. Numbers indicate an error ≥ 0.5%. (B) Analysis of the error associated with determining key fcyton cellular parameters. Box plots represent 5,25,50,75, and 95 percentile values. Outliers are not shown. For a comparison of all 12 parameters see Figure S1 (blue) and Figure S2 (purple).

Next, we integrated the two modules (Figure 1) and characterized the resulting performance. This integrated approach uses the best-fit cell fluorescence parameters to represent the cell population solutions as fluorescence histograms, enabling direct comparison to the experimental data, and obviating the need for an *ad hoc* objective function during population model fitting (compare Equations 28 and 29 in Text S1). After applying each approach to the panel of generated datasets, we calculated the generational average normalized percent count errors (Figure 4A), as well as parameter error distributions (Figure 4B). Both the sequential and integrated approaches resulted in relatively low generational cell count errors on average, however, the integrated approach outperformed sequential model fitting for predicting the generational cell counts at late time points (Figure 4A). The improvement was more readily apparent in the distribution of parameter fit errors: all parameter error distributions were shifted toward zero when the integrated rather than the sequential model fitting approach was used (p-values for each parameter distribution ≤1E-5, Mann-Whitney U test). In fact, all but the Tdie_1+_ parameter errors showed a very dramatic improvement (p-value ≤1E-10, Mann-Whitney U test). To determine if the improvement was due to a propagation of fit errors caused by sequential fitting steps, we compared both the sequential and integrated method when the population model was fitted to perfect counts or when perfect fluorescence parameters were used, respectively. (Figure S2) When comparing both approaches under ideal conditions, integrated fitting resulted in overall better cell count errors at later time points (Figure S2A.), and improved error distributions for fcyton parameters F_0_ and N (p-value ≤0.05, Mann-Whitney U test). Next, by comparing the integrated approach to individual computational modules, we found that the accuracy of the integrated approach was comparable to the accuracy associated with fitting the fcyton model cell counts to known counts using the *ad hoc* optimized objective function, as well as when the integrated method was used with known cell fluorescence parameters (Figure S2). This suggests that the integrated method minimizes the propagation of errors, as it is comparable to fitting to the original generated cell counts using a complex optimized objective function, and because eliminating the fluorescence model fitting error did not significantly improve the fit.

To develop best practices for employing integrated fitting, we examined how the number of experimental time points, the number of computational fit attempts, and selection of the objective function would affect fitting accuracy. We found that using the best of eight, three or one computational fit attempts decreased the average normalized generational cell count errors and asymptotically improved the distributions of parameter errors (Figure S3). Since choice of time points can also affect solution quality, we repeated our error analysis with fewer time points. While more frequent sampling improved the median and variance of the error distributions, key time points turned out to be those close to the start of the experiment, just when the first cell divisions have occurred, and when the founding generation has all but disappeared, affecting fcyton parameters F_0_, N, and Tdie_0_ to a higher degree (Figure S4). To test which objective function to use for integrated model fitting, we tested three objective functions of increasing complexity: simple mean sum of absolution deviations (MAD), mean root sum of squared deviations (MRSD), and mean root sum of squared deviations with Pearson correlation (MRSD+). We fitted sets of 1,000 generated time courses (see Methods) with each of the three objective functions (Figure S5B) and we calculated the generational average normalized percent count errors (Figure S5A), as well as parameter error distributions (Figure S5C). The results showed that using the MRSD+ objective function resulted in the lowest average normalized generation percent count errors, however all three objective functions resulted in comparable fcyton parameter error distributions (p-value>0.05, Mann-Whitney U test), except error in N for MAD was significantly higher compared to MRSD/MRSD+ (p-value <1E-10, Mann-Whitney U test).

Finally, we tested how the length of time needed to fit both of the models depends on the number of time points and cell generations used. As expected, the running time increased approximately linearly with the number of time points fitted and number of generations modeled, with typical time courses (9 generations, 7 time points) taking on average 2.11 minutes to fit (Table S1).

### Developing Solution Confidence and Comparison to the Most Recent Tool

**Figure 5:**
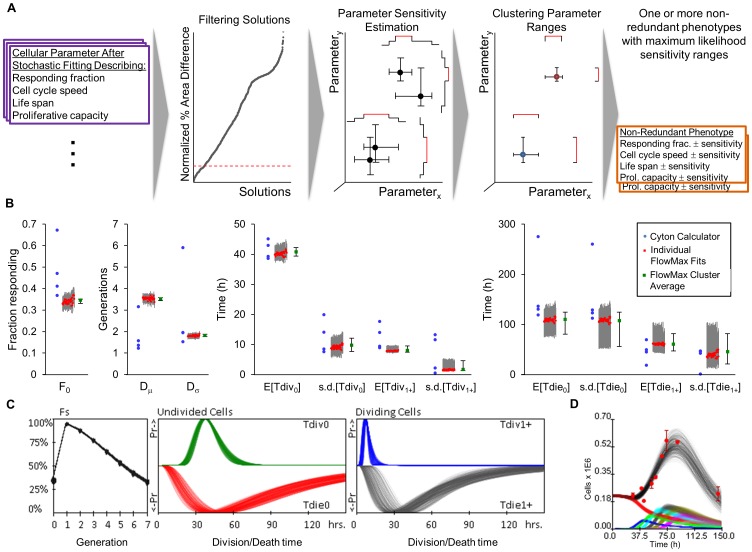
Comparison of FlowMax to the Cyton Calculator. The Cyton Calculator [9] and a computational tool implementing our methodology, “FlowMax,” were used to train the cyton model with log-normally distributed division and death times on a CFSE time course of wildtype B cells stimulated with lipopolysaccharides (LPS). The best-fit generational cell counts were input to the Cyton Calculator. (A) Visual summary of solution quality estimation pipeline implemented as part of FlowMax. Candidate parameter sets are filtered by the normalized % area difference score, parameter sensitivity ranges are calculated, parameter sensitivity ranges are clustered to reveal non-redundant maximum-likelihood parameter ranges (red ranges). Jagged lines represent the sum of uniform parameter distributions in each cluster. (B) Best fit cyton model parameters determined using the Cyton Calculator (blue dots) and our phenotyping tool, FlowMax (square red individual fits with sensitivity ranges represented by error bars and square green weighted cluster averages with error bars representing the intersection of parameter sensitivity ranges for 41 solutions in the only identified cluster). (C) Plots of Fs (the fraction of cells dividing to the next generation), and log-normal distributions for the time to divide and die of undivided and dividing cells sampled uniformly from best-fit cluster ranges in (B). (D) Generational (colors) and total cell counts (black) are plotted as a function of time for 250 cyton parameter sets sampled uniformly from the intersection of best-fit cluster parameter ranges. Red dots show average experimental cell counts for each time point. Error bars show standard deviation for duplicate runs.

As part of a crucial third step, we developed a computational pipeline for estimating both the sensitivity and redundancy of solutions. At the end of population model fitting, multiple candidate best-fit parameter sets are found (Figure 1, step 2). To enable objective evaluation of solutions, we estimate parameter sensitivities for candidate fits with particularly low ending objective function values and use an agglomerative clustering approach to combine pairs of candidate solutions until only disjoint clusters remain, representing non-redundant maximum-likelihood parameter ranges (Figure 5A and Text S1). To demonstrate the benefit of using our solution sensitivity and redundancy estimation procedure, we compared our approach to the most recent phenotyping tool, the Cyton Calculator [9]. The Cyton Calculator was designed for fitting the cyton model [2] to generational cell counts determined using flow cytometry analysis tools. The cyton model incorporates most of the key biological features of proliferating lymphocytes, with the exception that responding cells are subject to competing death and division processes. We demonstrated the utility of our method, by phenotyping a CFSE time course of wildtype B cells stimulated with bacterial lipopolysaccharides (LPS) with both the Cyton Calculator as well as FlowMax, a tool implementing our methodology. While several qualitatively good solutions were found using the Cyton Calculator for four different starting combinations of parameters (Table S2), we could not objectively determine if the best-fit solutions were representative of one solution with relatively insensitive parameters, or four unique solutions (Figure 5B blue dots). As a comparison, we repeated the fitting using FlowMax under identical fitting conditions (Figure 5B, red individual solutions and clustered averages in green). Best-fit clustered FlowMax cyton parameters yielded one unique quantitatively excellent average fit (3.01% difference in normalized percent histogram areas). The best-fit parameter ranges showed that the division times and the propensity to enter the first round of division are important for obtaining a good solution, while predicted death times can be more variable without introducing too much fit error (Figure 5C). Plotting cell count trajectories using parameters sampled uniformly from maximum-likelihood parameter sensitivity ranges revealed that while the early B cell response is constrained, the peak and late response is more difficult to determine accurately (Figure 5D).

### Investigating how data Quality Affects Solution Sensitivity and Redundancy

**Figure 6:**
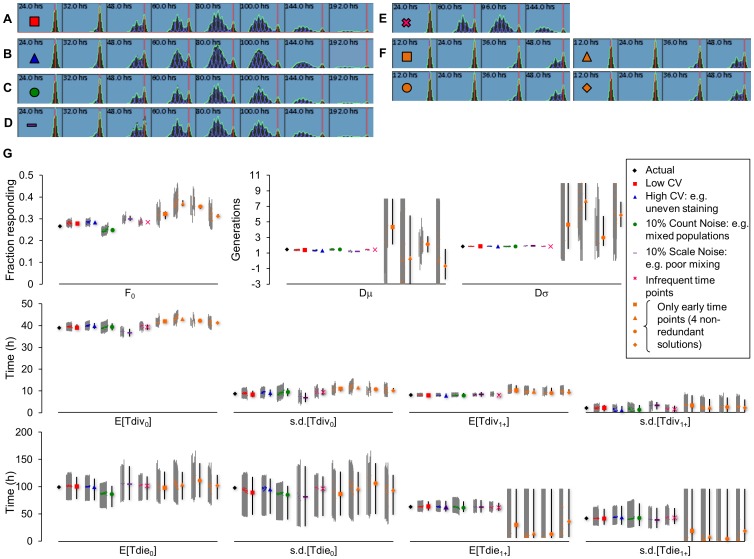
Testing the accuracy of the proposed approach as a function of data quality. Six typical CFSE time courses of varying quality were generated and fitted using our methodology (Figure 1). (A-F) The best-fit cluster solutions are shown as overlays on top of black histograms for indicated time points. Conditions tested were (A) low CV, (B) high CV (e.g. poor staining), (C) 10% Gaussian count noise (e.g. mixed populations), (D) 10% Gaussian scale noise (poor mixing of cells), (E) four distributed time points (e.g. infrequent time points), (F) four early time points from the first 48 hours (see Methods for full description). (G) Parameter sensitivity ranges for each solution in each non-redundant cluster next to the maximum likelihood parameter ranges are shown for fcyton fitting. The actual parameter value is shown first (black dot).

We tested how sources of imperfections in typical experimental CFSE data affected the outcome of our integrated fitting procedure. Starting with the best fit average wildtype B cell time course stimulated with bacterial lipopolysaccharides (LPS), we generated *in silico* CFSE datasets. Specifically, we wanted to test the effect of time point frequency, increased fluorescence CV (e.g. due to poor CFSE staining), increased Gaussian noise in generational counts (e.g. mixed populations), and increased Gaussian noise in the total number of cells collected during each time point (e.g. mixing/preparation noise) (Figure 6). For each generated dataset, we fitted cell fluorescence parameters, used the best-fit fluorescence parameters as adaptors during a subsequent 100 rounds of population model fitting, filtered poor solutions, calculated parameter sensitivities, and clustered the solution ranges to obtain maximum-likelihood non-redundant solution ranges (Figure 1).

Results show that increasing CV or using only four, albeit well positioned time points, does not significantly impact the quality of the fit, with all parameters still accurately recovered (blue triangles, pink crosses). On the other hand, adding random noise in the number of cells per peak or per time point results in increased error in fcyton parameters F_0_, Tdie_0_ and to a lesser degree s.d.[Tdiv_0_] and s.d.[Tdiv_1+_] (Figure 6 green circles and purple bars). However, only using early time points resulted in egregious errors with most parameters displaying diminished sensitivity and higher deviation from the actual parameter value. Indeed, our method identified four non-redundant solutions when fitting the early time point only time course (Figure 6, orange).

### Phenotyping B Lymphocytes Lacking NFκB Family Members

We next applied the integrated phenotyping tool, FlowMax, to a well-studied experimental system: the dynamics of B cell populations triggered by *ex vivo* stimulation with pathogen-associated molecular patterns (PAMPs) or antigen-receptor agonists. B cell expansion is regulated by the transcription factor NFκB, which may control cell division and/or survival. Indeed, mice lacking different NFκB family members have been shown to have distinct B cell expansion phenotypes in response to different mitogenic stimuli [19].

**Figure 7:**
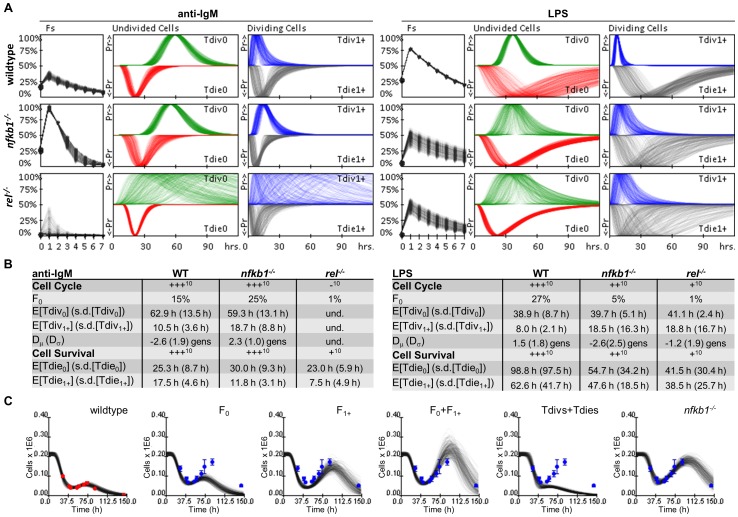
Phenotyping WT, *nfkb1^−/−^*, and *rel^−/−^* B cells stimulated with anti-IgM and LPS. (A) Visual summaries of best-fit phenotype clusters for WT (top), *nfkb1^−/−^* (middle), and *rel^−/−^* (bottom) genotypes stimulated with anti-IgM (left), and LPS (right). To visualize cellular parameter sensitivity, 250 sets of parameters were selected randomly from within parameter sensitivity ranges and used to depict individual curves for the fraction of responding cells in each generation (Fs) and lognormal distributions for time-dependent probabilities to divide (Tdiv) and die (Tdie) for undivided and divided cells. (B) Tables summarizing the best fit cellular parameters determined using the integrated computational tool, FlowMax, as well as the relative amount of cell cycling and survival reported in previous studies [12]. Values in parentheses represent the lognormal standard deviation parameters. (C) Total cell counts simulated with the fcyton model when indicated combinations of *nfkb1* ^−/−^specific parameters were substituted by WT-specific parameters during anti-IgM stimulation (“chimeric” solutions). Dots show WT (red) and *nfkb1* ^−/−^ (blue) experimental counts. Error bars show cell count standard deviation for duplicate runs.

Using published studies as a benchmark, we tested the utility of FlowMax. Using purified naïve B lymphocytes from WT, *nfkb1^−/−^*, and *rel^−/−^* mice, stained with CFSE, we obtained flow-cytometry data following LPS and anti-IgM stimulation over a six day time course. We then used FlowMax to arrive at the best-fit single-cell representation of the CFSE population data for each experimental condition tested (Figure 7A and Figure S6) and tabulated the cellular parameter values from the best family of clustered solutions for all conditions tested alongside our summary of the previously-published results (Figure 7B). The best-fit solution clusters fit the time courses well (11.95% median normalized percent area error), with the larger errors naturally biased toward weekly proliferating populations (Figure S6). Our analysis revealed that in response to anti-IgM cRel-deficient B cells are unable to enter the cell division program, as evidenced by a low F_0_ value. However, in response to LPS, *rel^−/−^* and *nfkb1^−/−^* B-cells show both cell survival and activation phenotypes, suggesting the involvement of other *nfkb1* functions downstream of the receptor TLR4 (Figure S7). These computational phenotyping results are in agreement with the conclusions reached in prior studies using traditional methods such as tritiated thymidine incorporation, as well as staining for DNA content or membrane integrity (propidium iodide) to measure cell population growth as well as the fractions of cycling and dying cells, respectively [11]. In particular, in response to LPS, the *nfkb1* gene product p105 (rather than p50) was shown to mediate B-cell survival via the Tpl2/ERK axis [12]. However, our results extend the published analysis by quantifying the contributions of the cell survival and decision making functions of these genes to B lymphocyte expansion. For example, whereas *nfkb1* and *rel* appear to equally contribute to cell cycle and survival, *rel* has a more critical role in the cellular decision to enter the cell division program (Figure 7 and Figure S7).

Interestingly, in response to anti-IgM, our analysis reveals a previously unknown suppressive role for *nfkb1* of limiting the number of divisions that cells undergo (Figure 7, compare D_μ_ and D_σ_). In response to LPS, Fs are reduced in *nfkb1* ^−/−^ B cells, but they are higher in response to anti-IgM. This affects mostly the later progressor fractions, e.g. F_1_, F_2_. To examine the contribution of each parameter type (decision making, cell cycle times, death times) we developed a solution analysis tool, which allows for model simulations with mixed knockout- and wildtype-specific parameters to illustrate which parameter or combination of cellular processes substantially contribute to the knockout phenotype. In the case of IgM-stimulated *nfkb1^−/−^*, this analysis reveals that the later cell decision parameters (e.g. F_1,2,…_) are necessary and largely sufficient to produce the observed phenotype (Figure 7C, Figure S7).

## Discussion

Recent advances in flow cytometry and mathematical modeling have made it possible to study cell population dynamics in terms of stochastic cellular processes that describe cell response, cell cycle, and life span. Interpreting CFSE dye dilution population experiments in terms of biologically intuitive cellular parameters remains a difficult problem due to experimental and biological heterogeneity on the cellular level. While available population models may be fitted to generational cell counts, a remaining challenge lies in determining the redundancy and size of the solution space, a requirement for developing confidence in the quantitative deconvolution of CFSE data. Developing a methodology for objective interpretation of CFSE data may lead to quantitative mechanism-oriented insights about cellular decision-making, and allow for improved and automated diagnosis of such data in the clinic.

In this study we present an integrated phenotyping methodology, exemplified by the computational tool FlowMax, which addresses these challenges. FlowMax comprises the tools needed to construct CFSE histograms from flow cytometry data, fit a fluorescence model to each histogram, determine sets of best fit cellular parameters that best describe the CFSE fluorescence time series, and estimate the sensitivity and redundancy of the best fit parameters (Figure 1). By using the cell fluorescence model to translate between generation-specific cell counts of the cell population model and the CFSE fluorescence profiles, the method ensures that the population dynamics model is trained directly on the experimental fluorescence data, without relying on *ad hoc* scoring functions. While our general methodology can be relatively easily adopted for use with any population dynamics and cell fluorescence models (including population models that incorporate both CFSE label and population dynamics [13], [16]–[18]), we adopted a version of the cyton model because it explicitly incorporates most features of proliferating lymphocytes in an intuitive manner, forms the basis of the Cyton Calculator tool, and could be easily adapted to include new observations from single-cell studies. While, the cyton model is over-determined and it is possible that minimal alternative models may describe the noisy CFSE data equally-well [7]. For example, it is possible that models with exponential distributions for the time to divide and die, or models which do not include generational dependence for division/death may be able to describe the data. However, independent studies have shown that lymphocyte cycling and programmed cell death show delay times and conform to log-normal distributions, and that the fraction of lymphocytes exiting the cell cycle as well as the timing for division and death of lymphocytes are generation-dependent [2], [3], [20]. Our attempts at fitting a typical experimental dataset using minimal models confirmed that to model B cell dynamics both a delay in division/death timing (e.g. using log-normal distributions) as well as distinguishing between generations (e.g. undivided/divided) is essential (unpublished data). Within FlowMax we chose to decouple treatment of cell fluorescence from population dynamics and allow for manual compensation for general fluorescence changes such as dye catabolism (See Text S2). Treating such experimental heterogeneity separately from biological variability was essential for computational tractability of solution finding via repeated fitting.

Fitting generated datasets allowed us to evaluate individual fitting steps, and when these were combined in an integrated or sequential manner. While, the cell fluorescence model is readily trained on the generated data, especially if multiple peaks are present (Figure 2B–C), not all fcyton model parameters are equally determinable, as parameters for Tdie_1+_ and Dμ were associated with significant median errors (Figure 3C and Figure S2). When both models were fitted, doing so in an integrated manner (using the fitted cell fluorescence parameters as adaptors during population model optimization) outperformed doing so sequentially in terms of both solution statistical significance (Figure 4A) and fcyton parameter error distributions (Figure 4B and Figure S1). This is not surprising as the integrated method avoids errors introduced during fluorescence model fitting, by optimizing the cell population model on the fluorescence histograms directly (Figure S2). Furthermore, by using the fluorescence model as an adaptor, contributions from each fluorescence intensity bin are automatically given appropriate weight during population model fitting, while the sequential approach must rely on *ad hoc* scoring functions to achieve reasonable, albeit worse, fits. The accuracy of the integrated fitting approach improves asymptotically with the number of fit points used (Figure S3), and is dependent on the choice of time points used, with errors in key fcyton model early F_0_, N, and late Tdie_0_ parameters especially sensitive to sufficiently early and late time points, respectively (Figure S4). Testing potential scoring functions demonstrated that while the methodology is relatively robust to specific objective function selection, an objective function including both a mean root sum of squared deviations as well as a correlation term resulted in lower errors in average fitted generational counts (Figure S5). Finally, fitting both the cell fluorescence and fcyton model typically requires only a few minutes on a modern computer (Table S1), suggesting that our methodology and tool can be used to process a long duplicate time course in about a day.

The analysis of our fitting methodology revealed a limit on the accuracy of fitted model parameters, even under idealized conditions of perfect knowledge of experimental heterogeneity and assuming the fcyton model is a perfect description of B cell dynamics (Figure 3), suggesting that objective interpretation requires solution sensitivity and redundancy estimation. We compared several qualitatively good model fits obtained with the Cyton Calculator [9] to our phenotyping tool FlowMax (Table S2 and Figure 5). Using the Cyton Calculator, best-fit parameter sets (Figure 5B blue dots) are subject to choice of initial parameters (Table S2). Repeated fitting with different fitting conditions yielded qualitatively good solutions with different parameter values. Conversely, the solution quality estimation integrated into our methodology (Figure 5A) revealed that only one set of parameters best describes the dataset, and that only a relatively small range of maximum-likelihood parameter values was common to good fits (Figure 5B green dots and ranges). Interestingly, most of the fitted parameters are in approximate quantitative agreement between the two methods, however, the maximum-likelihood parameter ranges determined by our methodology usually showed agreement with outlying parameter values determined by the Cyton Calculator, suggesting that picking a specific or average solution may be inappropriate (Figure 5B).

Testing how data quality affects solution redundancy and sensitivity reveals that the methodology is relatively robust to poor CFSE staining (high CV) as well as the frequency of time points used for fitting, assuming they are spaced throughout the time course (Figure 6). However, this is only true if time points are selected such that they capture the population behavior throughout the response, as picking only early time points resulted in global parameter insensitivity, degeneracy, and large parameter errors. Furthermore, poor mixing/preparation of cells (scale noise) or the presence of other cell populations (count noise) resulted in qualitatively good fits at the cost of some errors in perceived population parameters, highlighting the importance of fitting to two or more replicate time courses and working with a single cell type.

Finally, to demonstrate that our computational tool can provide valuable insights into the cellular processes underlying lymphocyte dynamics, we used FlowMax to phenotype B cells from NFκB-deficient mice, which show strong proliferative and survival phenotypes when stimulated with anti-IgM and LPS mitogenic signals (Figure S6). Our analysis of these cells confirmed the previously published data [11], [12] and extended the analysis to specific cellular processes in a quantitative manner. We found for example that the phenotype of *nfkb1^−/−^* and *rel^−/−^* is similar in the proliferation and survival of B-cells, except in the ability of resting B cells to exit the G_0_ stage, which is more critically controlled by *rel* gene product cRel (Figure 7A). This may reflect that while cRel is activated early and required for all aspects of B-cell proliferation, the *nfkb1* gene product p105 is thought to provide for lasting ERK1 activity [21] that may facilitate primarily later stages of B-cell proliferation. Furthermore, our analysis revealed a previously unappreciated anti-proliferative role for NFκB gene *nfkb1* during anti-IgM stimulation (Figure 7B). Although more subtle, this phenotype was revealed because we were able to distinguish between early pro-proliferative cellular processes (F_0_, Tdiv_0_, Tdie_0_) and later ones (F_1+_, Tdiv_1+_, Tdie_1+_), which may otherwise be overshadowed by early parameters that more prominently determine bulk population dynamics, but importantly determine the proliferative capacity of B cells. We confirmed the importance of the later parameters by modeling population dynamics with “chimeric” parameter sets derived from wildtype and knockout model fits (Figure 7C and Figure S7). How *nfkb1* may dampen late proliferative functions in response to anti-IgM but not LPS remains to be investigated. Preliminary results indicate that the *nfkb1* gene product p50, which may have repressive effects as homodimers, is actually less abundant following anti-IgM than LPS stimulation. Conversely the *nfkb1* gene product p105 is more abundant following anti-IgM than LPS stimulation and could inhibit signaling in two ways. Induced expression of p105 may block MEK1/ERK activation by Tpl2 [22], or it may function to provide negative feedback on NFκB activity, as a component of the inhibitory IκBsome complex [23], [24]. Future studies may distinguish between these mechanisms and examine the role of the IκBsome in limiting the proliferative capacity of antigen-stimulated B cells.

## Models and Methods

### Ethics Statement

Wildtype and gene-deficient *rel* and *nfkb1* mice were maintained in ventilated cages. Animal studies were approved by the Institutional Animal Care and Use Committee of the University of California, San Diego.

### Modeling Experimental Cell Fluorescence Variability

For the cell fluorescence model, we adopted a mixture of Gaussians model for representing log-fluorescence CFSE histograms. The mean, μ, and standard deviation, σ, for a Gaussian distribution of cellular fluorescence in a specific generation, g, is calculated as
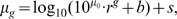
(1)




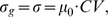
(2)where *r* represents the halving ratio (∼0.5), *b* the background (autofluorescence) [25], *s* is a shift parameter used to adjust the fluorescence of the whole distribution during fitting, and *CV* is the generation-invariant Gaussian coefficient of variation. While the CV is generation-invariant, fluorescence parameters are allowed to vary from time point to time point during fitting. These fluorescence parameters must be combined with generation-specific cell counts to describe a weighted fluorescence histogram that resembles typical CFSE data. Recent studies have shown that a mixture of Gaussians closely approximates experimental CFSE log-fluorescence histograms [9], [14], [15]. Our model is based on those suggested by Hodgkin et al [9]. In addition, Hasenauer *et al* suggest a mixture of log-normal distributions to approximate the combined heterogeneity in CFSE staining and autofluorescence [13]. A description of our model fitting strategy can be found in the Supplementary Methods (Text S1).

### Modeling Population Dynamics

For modeling population dynamics, we started with the generalized cyton model, which straightforwardly incorporates most biological features of lymphocyte proliferation [2], and forms the basis of the Cyton Calculator [9], the current state-of-the-art computational tool for interpreting CFSE-derived generational cell count data. To reflect the recent experimental finding that growing (i.e. responding) cells are resistant to death [3] we logically decoupled the division and death processes by explicitly removing the cell fate competition. In the so called, fcyton model, the fraction of responding cells in each generation (the Fs) control cell fate by ensuring that responding cells are protected from death, however the timing to the chosen fate (division or death) is still stochastically distributed. Specifically, the number of cells that divide and die for each cell generation, g, as a function of time, *t*, is found using
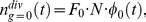
(3)

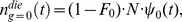
(4)

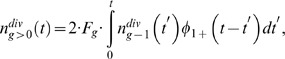
(5)

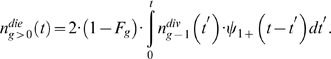
(6)


In equations (3–6) 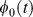, 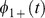, and 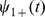 represent the cell age-dependent probability density functions that undivided cells will divide, divided cells will divide, undivided cells will die, and divided cells will die, respectively. The parameters *N* and 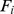, represent the starting cell count, and fraction of cells responding in generation i, respectively. The total number of cells, 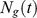 at time t and generation g is given by
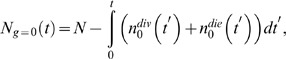
(7)

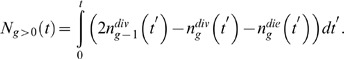
(8)


The progressor fractions, 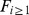, are calculated using a truncated Gaussian distribution similar to the “division destiny” curve suggested by Hawkins *et al* in the cyton model [2]:
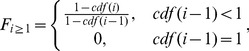
(9)where 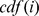 is the cumulative normal distribution with mean 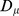 and standard deviation 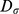. Since lymphocyte inter-division and death times are well-approximated by log-normal distributions [2], a total of 12 parameters are required to determine the cell count at any point in time in each generation: *N*, 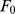, 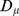, 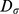, and eight parameters specifying the log-normal division and death distributions. For a full list of parameters and the ranges used during fitting, refer to Table S3. A description of our model fitting strategy can be found in the Supplementary Methods (Text S1).

### Testing Model Accuracy with Generated CFSE Fluorescence Time Courses

A total of 1,000 sets of randomized fcyton and fluorescence parameters within realistic ranges [2], [3], [9], [26], were generated (Table S3). The randomized fcyton parameters were applied to construct cell counts for eight generations ten time points up to192 hours (Table S4). The randomly chosen fluorescence parameters were then applied to construct weighted fluorescence histograms (Figure 2A). To test the accuracy of cell fluorescence model fitting, we trained the fluorescence model on the generated histogram time courses one histogram at a time. During fitting, peak weights were calculated analytically using a non-linear regression approach (see Text S1). Resulting best-fit model histogram areas under each peak were compared to their generated counterparts and the average percent errors of the counts normalized to the maximum generational count for each parameter set were plotted (Figure 2B, 3B, 4A, S1A, S2A, S3A, S4A, and S5A). To test the fcyton cell population model, we trained the model on known generational cell counts from the generated datasets. Resulting best-fit model generational counts and fcyton parameters were compared to their generated counterparts (Figure 2). To evaluate the accuracy of sequential model fitting, the generated datasets were used to first train the cell fluorescence model followed by a round of fcyton model fitting on the resulting best-fit generational cell counts using a simple squared deviation and a more complex *ad hoc* objective function (Figure 4(blue) and Figure S1). Next, the generated datasets were used to first train the cell fluorescence model followed by a round of fcyton model fitting to the fluorescence histograms using the best-fit cell fluorescence parameters to generate log-fluorescence histograms with peak weights determined by the population model, which were compared to generated histograms directly (proposed integrated fitting methodology). Different time point schedules were used when testing three or five time point time courses (see Table s4.). For demonstrating how data quality affects fitting of typical time courses, we used the fitted experimental wildtype LPS cluster solutions to generate six separate *in silico* time courses: a low CV time course (8 time points, CV = 0.18, ratio = 0.5, background = 100,shift = 0), a high CV time course (8 time points, CV = 0.23, ratio = 0.5, background = 100, shift = 0), a generation count noise time course (8 time points, CV = 0.18, ratio = 0.5, background = 100, shift = 0, each peak count scaled randomly by 1+N(μ = 0,σ = 0.1)), a scaled noise time course (8 time points, CV = 0.18, ratio = 0.5, background = 100, shift = 0, number of cells in histogram scaled randomly by 1+N(μ = 0,σ = 0.1)), an infrequent time point time course (4 time points from 24–144 h, CV = 0.18, ratio = 0.5, background = 100, shift = 0), and an early time point time course (4 time points from 12–48 h, CV = 0.18, ratio = 0.5, background = 100, shift = 0). Each time course was fitted 100 times using our full methodology (Figure 1), and parameter solution clusters were plotted (Figure 6). Refer to Table S4 for specific time point schedules used. Model fitting procedures are described in Text S1.

### Developing Measures of Confidence for Parameter Fits

We implemented a computational pipeline for estimating the redundancy and sensitivity of model solutions (Figure 1 step 3). A stochastic simulated annealing fitting procedure [27] was used to determine multiple best-fit solutions with random initial parameters (see Text S1). Next, we used a normalized percent area error (NPAE) metric for solution quality estimation which ranges between 0% and 100% difference in histogram areas:
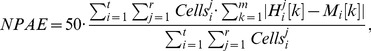
(10)where i and j represent time point i, and experimental run j, and *Cells*, *H,* and *M* represent total cell counts, experimental discrete histogram density, and model discrete histogram density with m total bins, respectively. Solution candidates with NPAE within 0.1 of the top were kept for quality estimation:
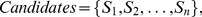
(11)where 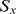 represents the xth set of best-fit parameters. These fits were subjected to one-dimensional parameter sensitivity estimation, which establishes an upper and lower bound on each parameter value that would result in the weighted percent histogram area error (NPAE) to, increase by 1 (1% normalized area difference increase), yielding two sets of sensitivity values for each parameter:
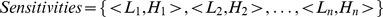
(12)where 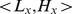 represents a 2-tuple consisting of sets of lower and upper parameter sets for 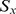, respectively (see Text S1). Since more than one non-redundant set of parameters may exist, we developed an agglomerative clustering algorithm which is designed to combine clusters with the highest parameter sensitivity overlap, arriving at sets of non-redundant maximum likelihood parameter ranges (see Text S1 for motivation and notes). Briefly, the solutions are clustered by continually agglomerating pairs of clusters 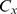,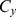 with highest total normalized overlap 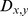 between parameters:



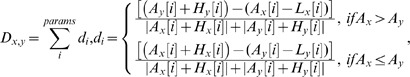
(13)where 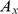 and 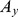
are weighted parameter averages for clusters 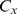 and 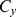, respectively. The agglomerated parameter sensitivity ranges are defined to be the intersection of ranges supported by all candidate solutions in the cluster, resulting in increasingly tighter estimates of the maximum likelihood parameter sensitivity ranges as more solutions are incorporated into the cluster. Clustering is terminated when cluster pairs for which parameter ranges are overlapping for all parameters no longer exist. When clustering parameter ranges, we keep track of a weighted average value that is guaranteed to be within the overlap between ranges being clustered, however its position is weighted according to the relative maximum distance from the average of each of the starting cluster averages:
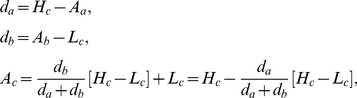
(14)where the distance (d), high(H), average (A), and low (L) values are used to agglomerate clusters a and b into cluster c and letting 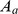
<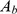. Finally, since solution clusters represent linear independent combinations of parameters, solution clusters are sampled uniformly (n = 1,000) within the clustered maximum- likelihood parameter ranges for all parameters simultaneously and clusters with median NPAE within 1% of the top cluster’s NPAE are kept to ensure that unrealistic parameter combinations were removed. Algorithms and motivation for sensitivity analysis and clustering are detailed in the supplement (Text S1).

### Comparing FlowMax to the Cyton Calculator

We used counts derived after fitting the cellular fluorescence model to the experimental wildtype B cell proliferation time courses stimulated with LPS (Figure S6), to repeatedly fit the cyton model using the Cyton Calculator [9] and compared to results from fitting the cyton model using FlowMax, a tool that implements our methodology and solution quality estimation procedure (Figure 5A). For the Cyton Claculator we used counts derived from fitting the cellular fluorescence model as input, while for FlowMax, we used the fluorescence data directly. To find Cyton Calculator solutions, we carried out Cyton Calculator fitting multiple times using varied starting parameters values sampled from ranges in Table S3, as suggested. Most-parameter combinations yielded qualitatively poor fits (determined visually by comparing total and generation cell counts to experimental data), and were discarded. Four qualitatively good solutions, determined visually by comparing total and generational cell counts to experimental data, were found using starting parameters listed in Table S2 (Figure 5B, blue dots). Using FlowMax involved 1,000 fits, automated solution filtering, parameter sensitivity estimation, and solution clustering. This allowed visualization of a family of solutions sampled from the maximum-likelihood sensitivity ranges for the only solution cluster identified.

### Testing how our Methodology is Affected by the Choice of Objective Function

To analyze how our methodology is affected by choice of objective function during fitting, we used 1,000 generated time courses to fit the fcyton model using best-fit cell fluorescence parameters as adaptors (our proposed integrated methodology). We tested three objective functions for comparing the model histograms to generated histograms: a simple mean sum of absolute deviations (MAD):
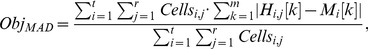
(15)a mean root sum of squared deviations (MRSD) objective function:
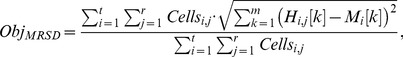
(16)and a mean root sum of squared deviations with Pearson correlation (MRSD+) objective function:



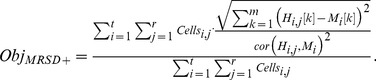
(17)In the above equations, 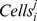 is the total cell count in run j for time point i, and cor(x,y) represents the Pearson correlation coefficient between the experimental histogram, 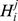, and modeled histogram, 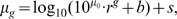. See also Figure S5 and Text S1.

### Generating Chimeric Solutions from Two Phenotypes

To dissect the contributions of several components of complex phenotypes, we used two sets of parameters (i.e. wildtype and mutant) and generated a “chimeric” set of parameters with combinations of F_0_, F_1+_ (Dμ, Dσ), Tdivs (E[Tdiv_0_], s.d.[Tdiv_0_],E[Tdiv_1+_], s.d.[Tdiv_1+_]), and Tdies (E[Tdie_0_], s.d.[Tdie_0_],E[Tdie_1+_], s.d.[Tdie_1+_]), copied from either set. The generated “chimeric” phenotypes were visualized (see below) and qualitatively compared to visualizations from the two originating phenotypes. In the case of *nfkb1* ^−/−^ anti-IgM stimulated B cells, this analysis confirmed that misregulation of the late progressor fractions (F_1+_) constituted the primary phenotype (Figure 7C).

### Visualizing Solution Clusters

Solution clusters were defined as sets of maximum-likelihood parameter sensitivity ranges that are overlapping between all solutions in a cluster (see Text S1). To visualize these solutions, parameter sets were sampled uniformly from within the clustered maximum-likelihood parameter sensitivity ranges independently for each parameter. For parameter visualization, the sampled parameters were used to plot the four lognormal distribution probability density functions (Tdiv_0_, Tdie_0_, Tdiv_1+_, Tdie_1+_), normalizing by the maximum probability per distribution. The fraction of responding cells in each generation (Fs) are plotted using connected dots on a scale between 0 and 1 for each generation (x axis), with the larger dot representing the independent F_0_ parameter (Figure 7). For population count visualization, the sampled parameter values were used to calculate cell count time series data by solving the fcyton model with the sampled parameters (Figure 7C and Figure S7). FlowMax provides options for plotting either the sampled solutions or the best-fit solutions found during model fitting. The best-fit cluster average solution (see also Text S1) is shown as an overlay for each experimental dataset (Figure S6).

### Using FlowMax to Phenotype CFSE Time Courses

We used a computational tool, which implements all of the steps for fitting experimental CFSE B cell datasets. A succinct tutorial is included in the supplementary text (Text S2). In brief, we used our computational tool to construct log-fluorescence CFSE histograms of viable B cells from raw CFSE data (see experimental methods below). For each log fluorescence histogram, the average fluorescence of undivided cells was selected manually based on previous time points. Then the cell fluorescence parameters were automatically determined for each time course subject to user constraints for the coefficient of variation, background autofluorescence, and die halving ratio, and shift of the undivided peak as well as an estimate of the maximum number of generations to be fitted to each time course (The default is set to eight [9]).The fitted cell fluorescence parameters were then used during the population dynamics fitting step to represent generational cell counts derived from the fcyton model. The population dynamics fitting step was repeated 1,000 times, poor results were removed from consideration, parameter sensitivity ranges were calculated (see Supplementary Methods in Text S1) and solutions were clustered to estimate solution redundancy (see Supplementary Methods in Text S1). The resulting best-fit families of solutions (determined by average error in histogram area sampled from parameter sensitivity ranges) for each experimental condition were compared.

### Experimental Methods

Primary splenocytes were isolated from 6–8 week old mice, naïve B cells purified using magnetic bead separation (Miltenyi Biotec), labeled with 4 µM 5(6)-Carboxyfluorescein diacetate, N-succinimidyl ester (CFSE) dye (Axxora) for 5 minutes at room temperature, and stimulated with 10 µg/mL LPS (Sigma) or 10 µg/mL goat anti-mouse IgM (Jackson Immunoresearch Inc.) B cells were grown in fresh media with 1% penicillin streptomycin solution (Mediatech Inc.), 5 mM L-glutamine (Mediatech Inc.), 25 mM HEPES buffer (Mediatech Inc.), 10% FCS and 2 µL/500 mL BME (Fisher Scientific) at a concentration of 2.5×10^5^ cells/mL in 48 well plates at 37°C for a period of 6 days.Cells were removed from media, stained with 10 ng/mL propidium iodide, and measured using an Accuri C6 Flow Cytometer (Accuri Inc.) at 28, 40, 43, 54, 59.5, 67.5, 74.5, 89, and 145 hours post stimulation. CFSE histograms were constructed after software compensation for fluorescence spillover and manual gating on viable (PI-negative) B cells using the FlowMax software. All measurements were performed in duplicate (B cells from the same spleen were cultured in separate wells, two wells per time point to ensure that each time course represented a single population of cells subject to only experimental variability).

## Supporting Information

Figure S1 **Accuracy of fitting the population model to generated fitted generational cell counts.** The simple squared deviation (grey) and *ad hoc* optimized (blue) scoring functions were used to fit the fcyton model to fitted generational cell counts for 1,000 sets of randomly generated CFSE time courses with parameters sampled uniformly from ranges in Table S3, and evaluated at times described in Table S4. (A) Average percent error in fitted generational cell counts normalized to the maximum generational cell count for each generated time course. Numbers indicate an error ≥ 0.5%. (B) Analysis of the error associated with determining all fcyton cellular parameters. Box plots represent 5, 25, 50, 75, and 95 percentile values. Outliers are not shown.
(TIF)

Figure S2 **Comparison of the integrated model fitting approach to training each model independently.** A collection of 1,000 randomly generated sets of CFSE time courses was used to analyze the errors associated with training the cell fluorescence model only (red), training the fcyton model on known cell counts (green), training the fcyton model using the known (orange) or fitted (purple) cell fluorescence parameters as adaptors during fcyton population model fitting. See also Tables S3, and S4. (A) Average percent error in fitted generational cell counts normalized to the maximum generational cell count for each generated time course. Numbers indicate an error ≥ 0.5%. (B) Analysis of the error associated with determining all fcyton cellular parameters. Box plots represent 5, 25, 50, 75, and 95 percentile values. Outliers are not shown.
(TIF)

Figure S3 **Analysis of the phenotyping accuracy as a function of the number of fit attempts (trials).** For each experiment, 1,000 CFSE time courses were generated with model parameters within ranges described in Table S3 and times described in Table S4. Generated time courses were used to fit the fcyton population model using the fitted cell fluorescence parameters as adaptors, using the best of one (light), three (medium), or eight (dark) fit trials. (A) Average percent error in fitted generational cell counts normalized to the maximum generational cell count for each generated time course. Numbers indicate an error ≥ 0.5%. (B) Analysis of the error associated with determining all fcyton cellular parameters. Box plots represent 5, 25, 50, 75, and 95 percentile values. Outliers are not shown.
(TIF)

Figure S4 **Analysis of the fitting accuracy when using fewer experimental time points.** For each experiment, three (light), five (medium), or ten (dark) time points were considered from a collection of 1,000 generated CFSE time courses with parameters sampled uniformly from ranges in Table S3, and evaluated at times described in Table S4. Generated time courses were then phenotyped using the integrated computational method (cell fluorescence parameters used as adaptors during fcyton fitting). (A) Average percent error in fitted generational cell counts normalized to the maximum generational cell count for each generated time course. Numbers indicate an error ≥ 0.3%. (B) Box plots represent 5, 25, 50, 75, and 95 percentile error values. Outliers are not shown.
(TIF)

Figure S5 **Analysis of the fitting accuracy as a function of objective function choice.** For each experiment, a mean absolute deviation (Obj_MAD_, light), a mean root square deviation (Obj_MRSD_, medium), and a mean root square deviation with correlation (Obj_MRSD+_, dark) were used to phenotype a collection of 1,000 generated CFSE time courses with parameter sampled uniformly from ranges in Table S3, and evaluated at times described in Table S4, using the integrated computational method (cell fluorescence parameters used as adaptors during fcyton fitting). (A) Average percent error in fitted generational cell counts normalized to the maximum generational cell count for each generated time course. Numbers indicate an error ≥ 0.5%. (B) Mathematical description of the objective functions used. (C) Analysis of the error associated with determining all fcyton cellular parameters. Box plots represent 5, 25, 50, 75, and 95 percentile values. Outliers are not shown.
(TIF)

Figure S6 **Best-fit fcyton solution overlays for stimulated wildtype,** ***nfkb1^−/−^*** **, and** ***rel^−/−^*** **B cell CFSE time courses.** CFSE fluorescence data was collected and phenotyped using FlowMax, a computational tool that implements our integrated methodology. Green overlays show the weighted average best-fit model solutions for six duplicate log-fluorescence CFSE time courses (filled histograms). Columns represent individual time points. Histograms are normalized to the highest count for each time course across experimental duplicates. X-axes are in log-fluorescence units and automatically chosen to encompass all fluorescence values across all time-points and experimental runs. Red line shows manually selected position of the undivided population. Times of collection are indicated next to each histogram. Background indicates stimulus (blue = LPS, purple = anti-IgM). See also Figure 7.
(TIF)

Figure S7 **Using chimeric model solutions to identify key fcyton parameters.** Total model cell counts determined when combinations of best-fit wildtype parameters were replaced by *nfkb1* ^−/−^ -specific (rows 1 and 3) and *rel^−/−^*specific (rows 2 and 4) best-fit maximum-likelihood parameter ranges for anti-IgM (rows 1 and 2) and LPS (rows 3 and 4) stimulation. Dots show wildtype (red) and knockout (blue) experimental counts. Error bars show standard deviation of cell counts from duplicate runs. Poor fitting indicates that the indicated parameters do not sufficiently describe the mutant phenotype.
(TIF)

Table S1 **Analysis of fit running time dependence on the number of time points and generations.** The average running time for fitting the cell fluorescence followed by fitting the fcyton cell population model using the best-fit cell fluorescence parameters to 300 generated time courses with four, seven, and ten time points is shown. Fitting was carried out using an assumed 6, 9, or 12 generations during fitting. Times are in minutes and errors are SEM. See also Table S3 and S4.
(DOCX)

Table S2 **Starting and fitted cyton model parameters for four successful Cyton Calculator fitting trials.** Starting cyton model parameter values that resulted in successful fits of our CFSE LPS-stimulated wildype B cell time course (columns 2–5) were chosen manually within ranges specified in Table S3. Corresponding Cyton Calculator [9] best-fit parameters are shown in columns 6–9. The data for experimental replicates is shown in Figure S6 (WT LPS).
(DOCX)

Table S3 **Cell fluorescence and population parameter ranges used to generate realistic CFSE time courses.** Selected ranges were chosen to exclude biologically implausible scenarios. Parameters were sampled evenly from the specified ranges whenever generating 1,000 time courses. The standard deviation parameters for the log-normal distributions: Tdiv_0_, Tdiv_1+_, Tdie_0_, Tdie_1+_ were further restricted to be less than or equal to their corresponding log-normal expected value parameters (e.g s.d[Tdiv_0_] ≤ E[Tdiv_0_]). Model fitting was restricted within these parameter ranges. Refer to Table S4 for the specific time points used.
(DOCX)

Table S4 **Time points considered for analysis of generated time courses.** For generated time courses, model solutions were sampled according to these time course schedules. Three, five, and ten time points were used in Figure S4. Four, four early, and eight time points were used in Figure 6. Four, seven, and ten time points were used when generating Table S1. Otherwise 10 time points were sampled from generated datasets. See also Table S3.
(DOCX)

Text S1 **Supplementary Methods.** This text includes notes and method for: description of CFSE time courses, fitting the cell fluorescence model, peak weight calculations during cell fluorescence model fitting, fitting the fcyton model to cell counts derived from fluorescence histograms, fitting the fcyton models to fluorescence histograms directly, parameter sensitivity estimation, and clustering by sensitivity agglomeration.
(DOC)

Text S2 **Succinct FlowMax tutorial.** This text describes the typical steps required to build CFSE log-fluorescence histograms from raw fcs datasets, apply the integrated fitting methodology, and interpret the results.
(DOC)
