# Supplementary figures and images for: FlowMax: A Computational Tool for Maximum Likelihood Deconvolution of CFSE Time Courses
Source: PLoS One. 2013 Jun 27;8(6):e67620. doi: 10.1371/journal.pone.0067620 (PMC3694893; doi:10.1371/journal.pone.0067620)

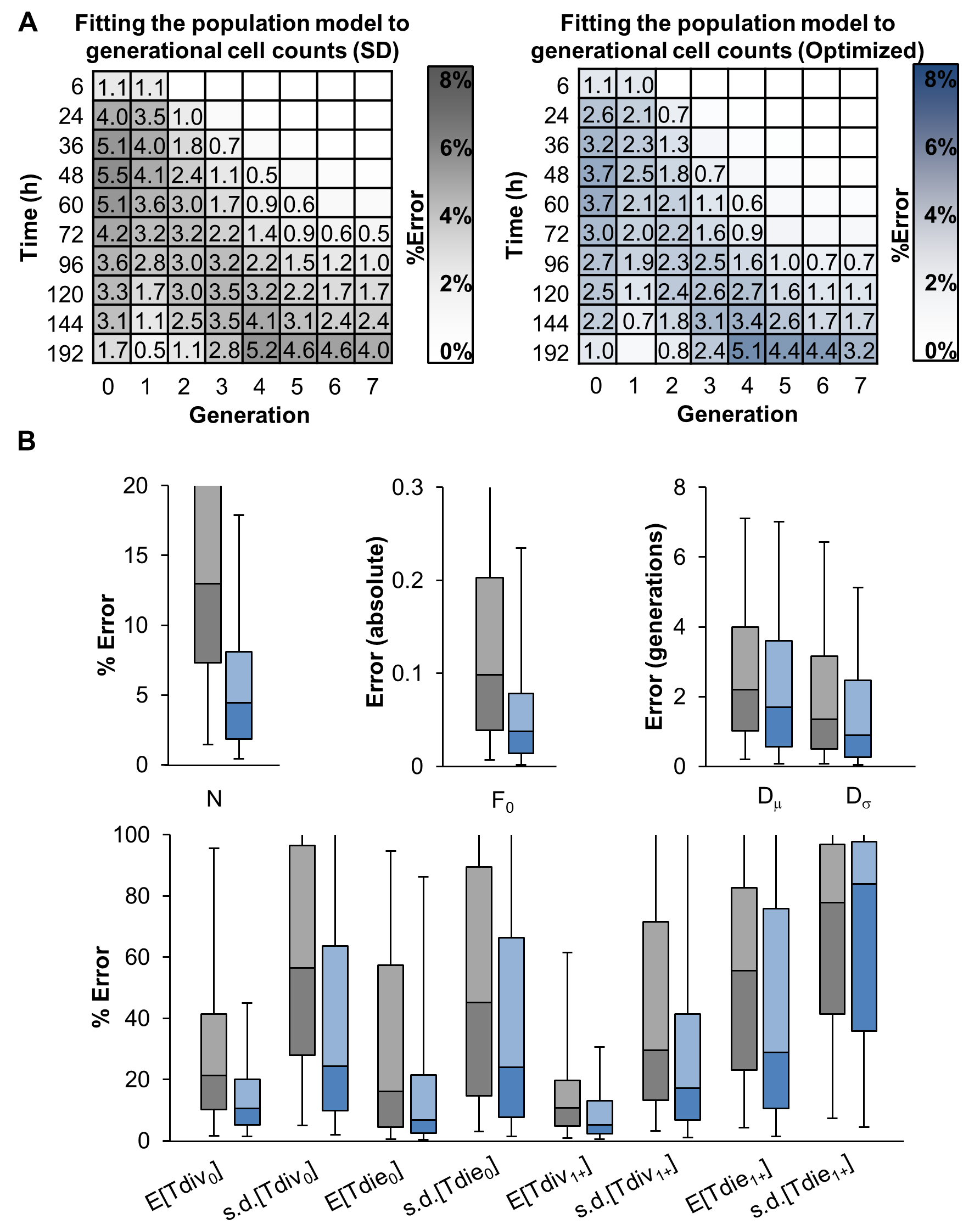

Supplement: Figure S1 — Accuracy of fitting the population model to generated fitted generational cell counts. The simple squared deviation (grey) and ad hoc optimized (blue) scoring functions were used to fit the fcyton model to fitted generational cell counts for 1,000 sets of randomly generated CFSE time courses with parameters sampled uniformly from ranges in Table S3, and evaluated at times described in Table S4. (A) Average percent error in fitted generational cell counts normalized to the maximum generational cell count for each generated time course. Numbers indicate an error ≥ 0.5%. (B) Analysis of the error associated with determining all fcyton cellular parameters. Box plots represent 5, 25, 50, 75, and 95 percentile values. Outliers are not shown. (TIF) [file pone.0067620.s001.tif]

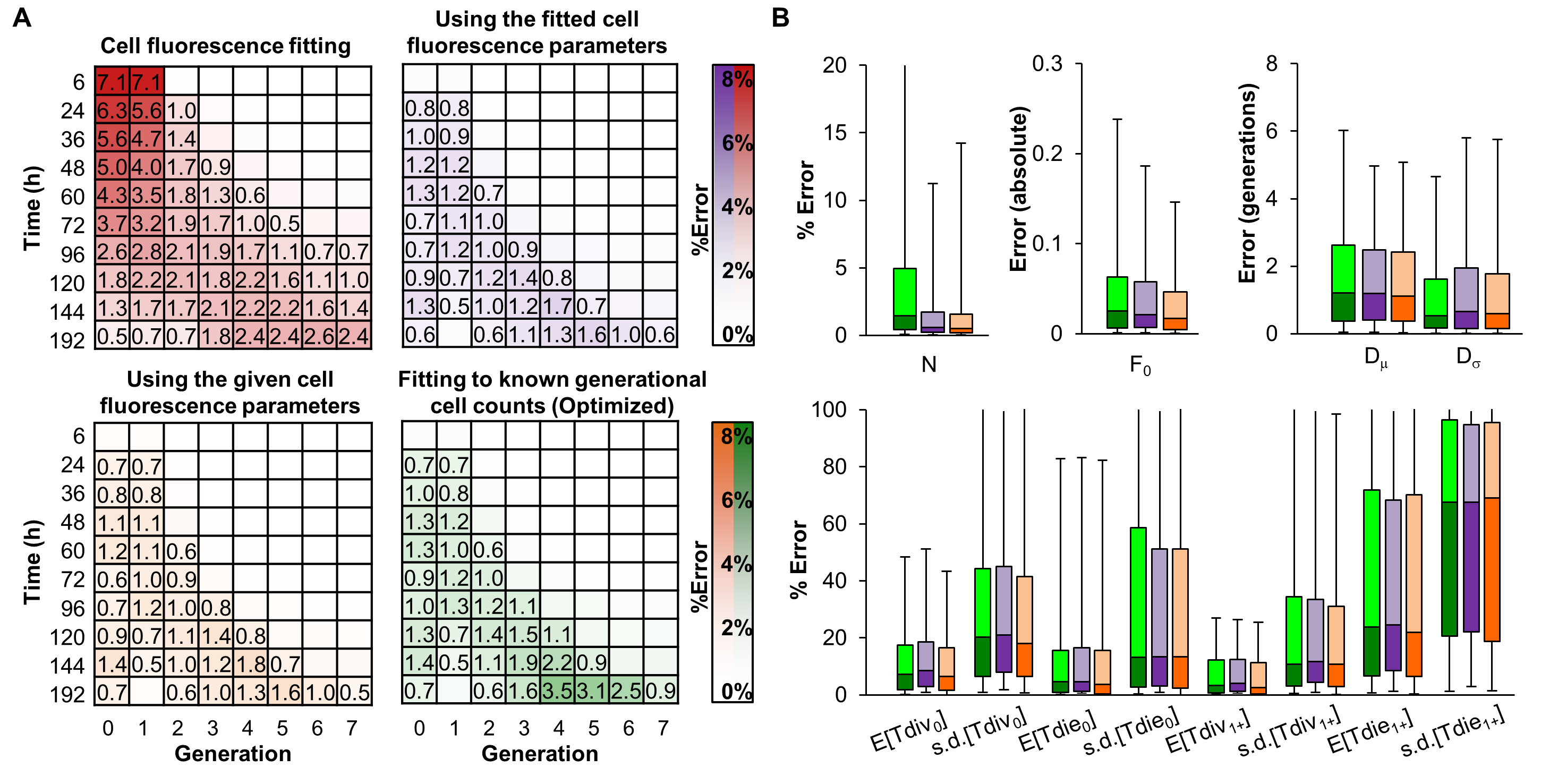

Supplement: Figure S2 — Comparison of the integrated model fitting approach to training each model independently. A collection of 1,000 randomly generated sets of CFSE time courses was used to analyze the errors associated with training the cell fluorescence model only (red), training the fcyton model on known cell counts (green), training the fcyton model using the known (orange) or fitted (purple) cell fluorescence parameters as adaptors during fcyton population model fitting. See also Tables S3, and S4. (A) Average percent error in fitted generational cell counts normalized to the maximum generational cell count for each generated time course. Numbers indicate an error ≥ 0.5%. (B) Analysis of the error associated with determining all fcyton cellular parameters. Box plots represent 5, 25, 50, 75, and 95 percentile values. Outliers are not shown. (TIF) [file pone.0067620.s002.tif]

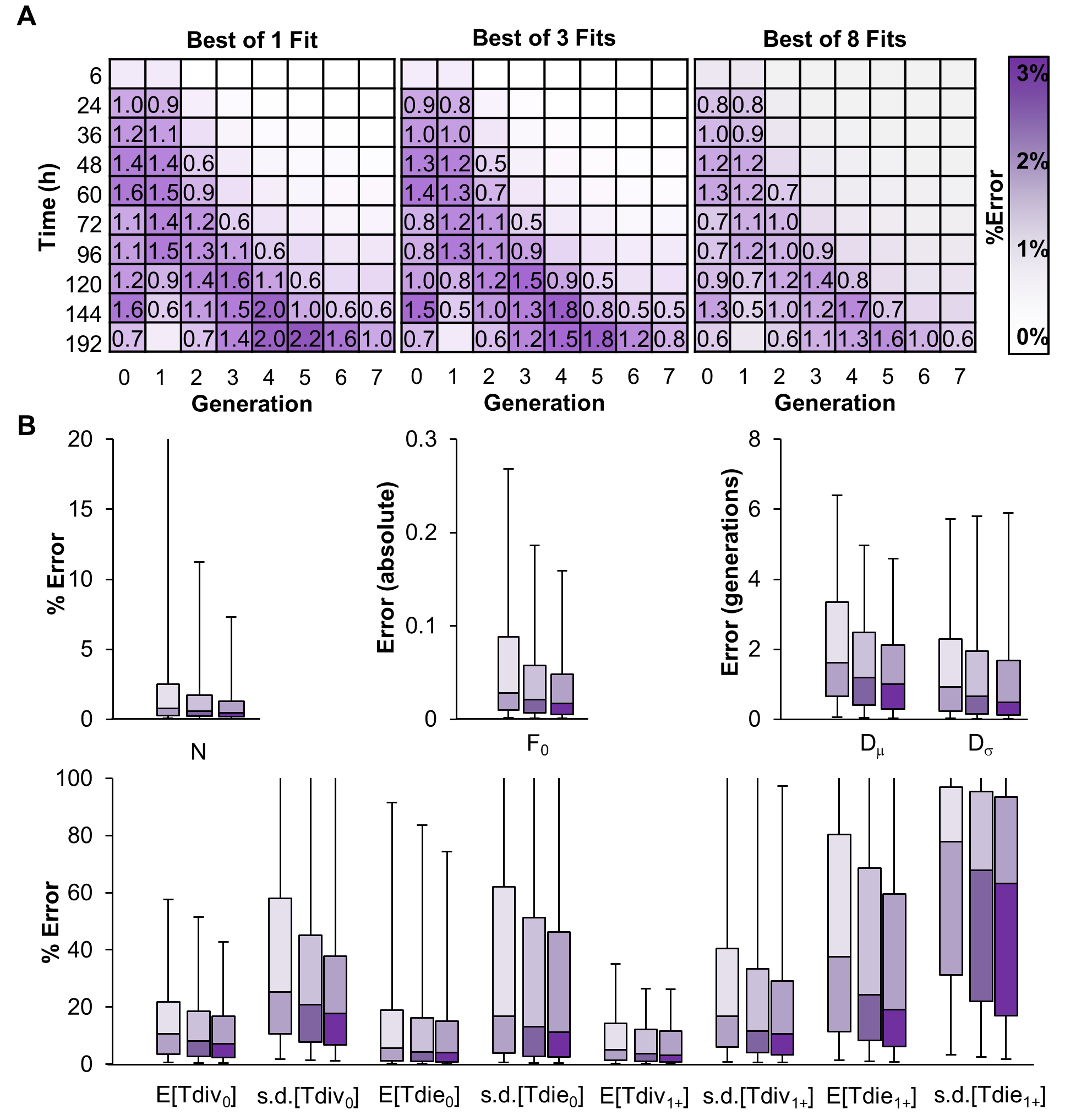

Supplement: Figure S3 — Analysis of the phenotyping accuracy as a function of the number of fit attempts (trials). For each experiment, 1,000 CFSE time courses were generated with model parameters within ranges described in Table S3 and times described in Table S4. Generated time courses were used to fit the fcyton population model using the fitted cell fluorescence parameters as adaptors, using the best of one (light), three (medium), or eight (dark) fit trials. (A) Average percent error in fitted generational cell counts normalized to the maximum generational cell count for each generated time course. Numbers indicate an error ≥ 0.5%. (B) Analysis of the error associated with determining all fcyton cellular parameters. Box plots represent 5, 25, 50, 75, and 95 percentile values. Outliers are not shown. (TIF) [file pone.0067620.s003.tif]

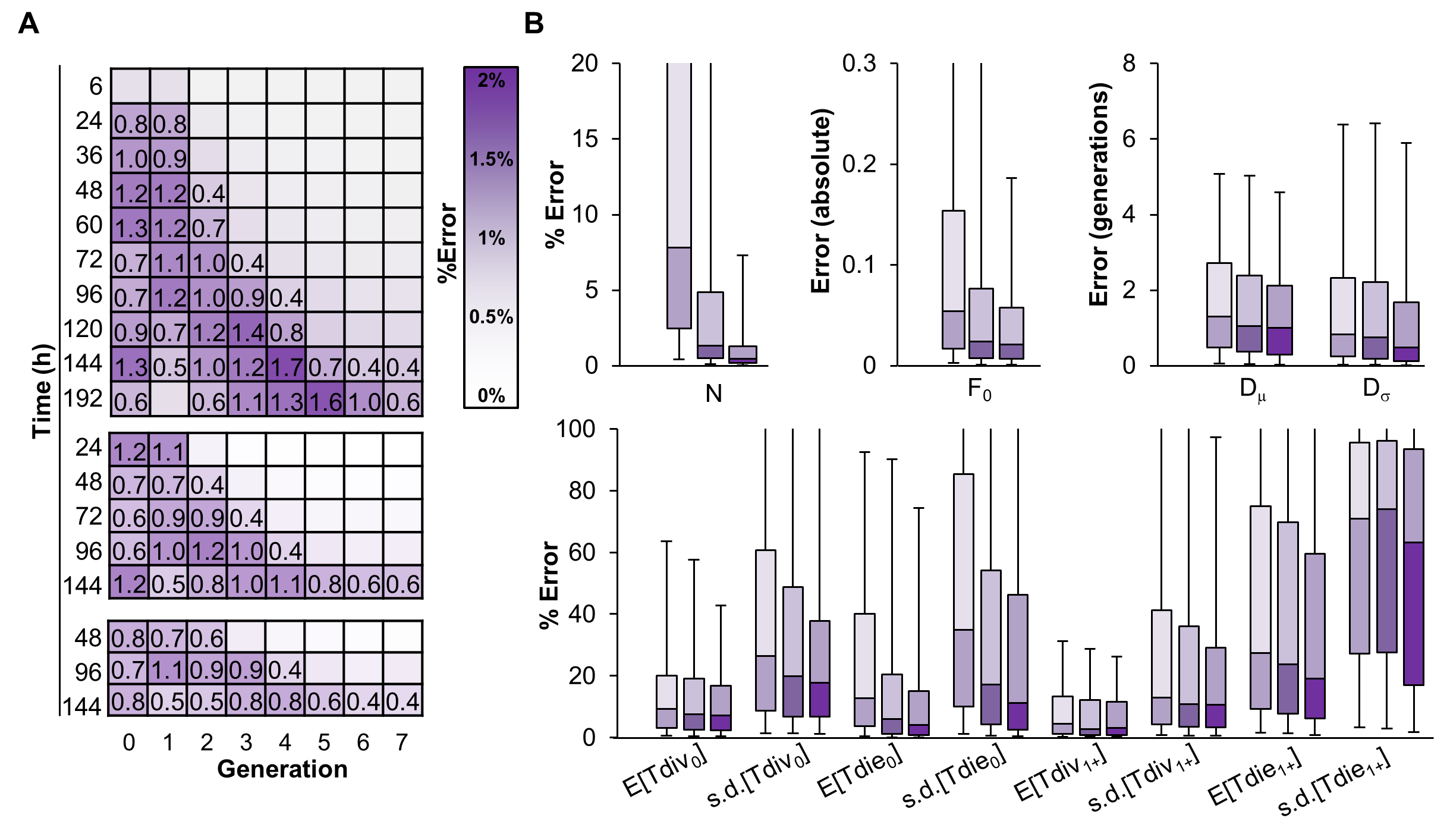

Supplement: Figure S4 — Analysis of the fitting accuracy when using fewer experimental time points. For each experiment, three (light), five (medium), or ten (dark) time points were considered from a collection of 1,000 generated CFSE time courses with parameters sampled uniformly from ranges in Table S3, and evaluated at times described in Table S4. Generated time courses were then phenotyped using the integrated computational method (cell fluorescence parameters used as adaptors during fcyton fitting). (A) Average percent error in fitted generational cell counts normalized to the maximum generational cell count for each generated time course. Numbers indicate an error ≥ 0.3%. (B) Box plots represent 5, 25, 50, 75, and 95 percentile error values. Outliers are not shown. (TIF) [file pone.0067620.s004.tif]

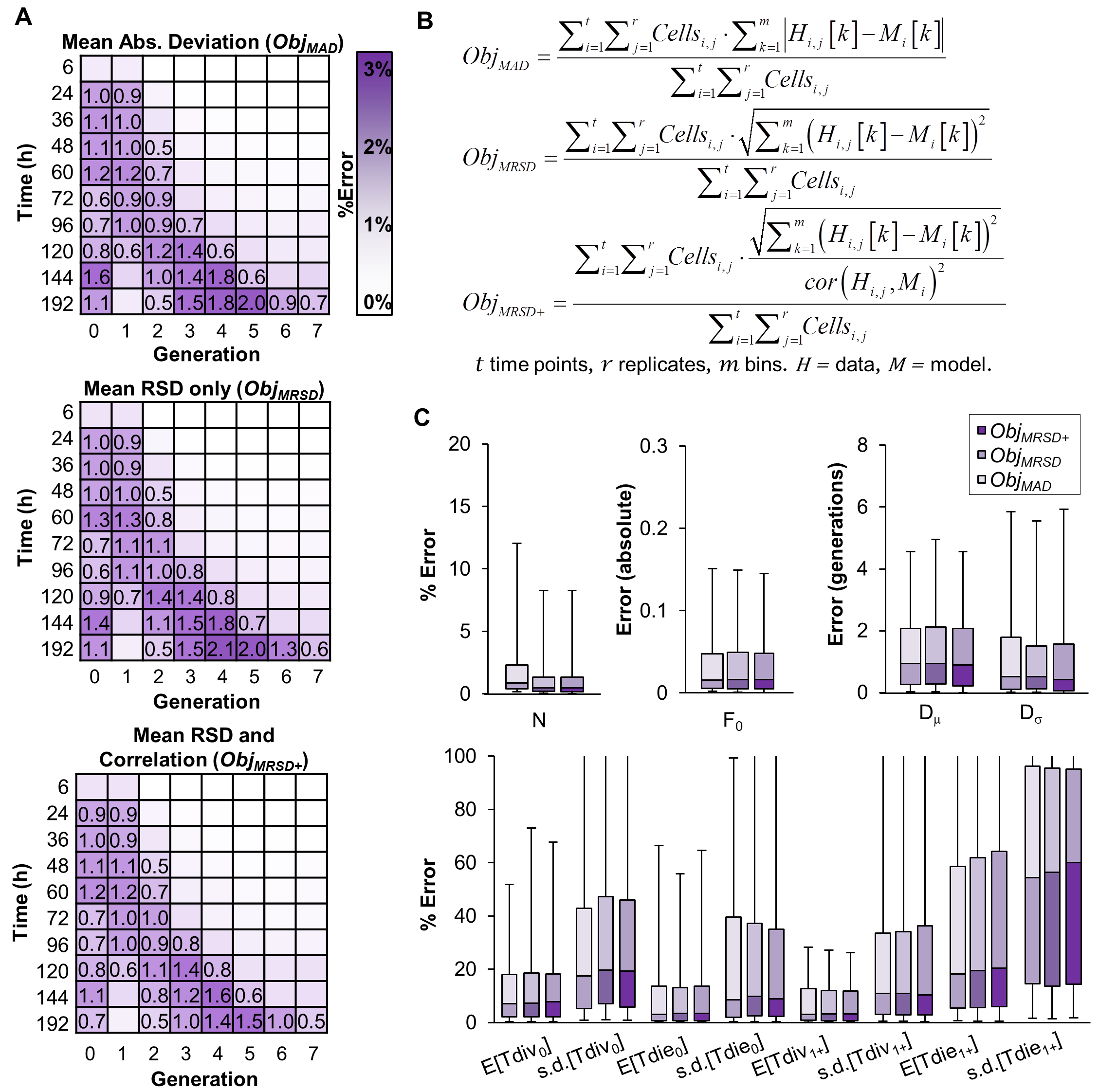

Supplement: Figure S5 — Analysis of the fitting accuracy as a function of objective function choice. For each experiment, a mean absolute deviation (ObjMAD, light), a mean root square deviation (ObjMRSD, medium), and a mean root square deviation with correlation (ObjMRSD+, dark) were used to phenotype a collection of 1,000 generated CFSE time courses with parameter sampled uniformly from ranges in Table S3, and evaluated at times described in Table S4, using the integrated computational method (cell fluorescence parameters used as adaptors during fcyton fitting). (A) Average percent error in fitted generational cell counts normalized to the maximum generational cell count for each generated time course. Numbers indicate an error ≥ 0.5%. (B) Mathematical description of the objective functions used. (C) Analysis of the error associated with determining all fcyton cellular parameters. Box plots represent 5, 25, 50, 75, and 95 percentile values. Outliers are not shown. (TIF) [file pone.0067620.s005.tif]

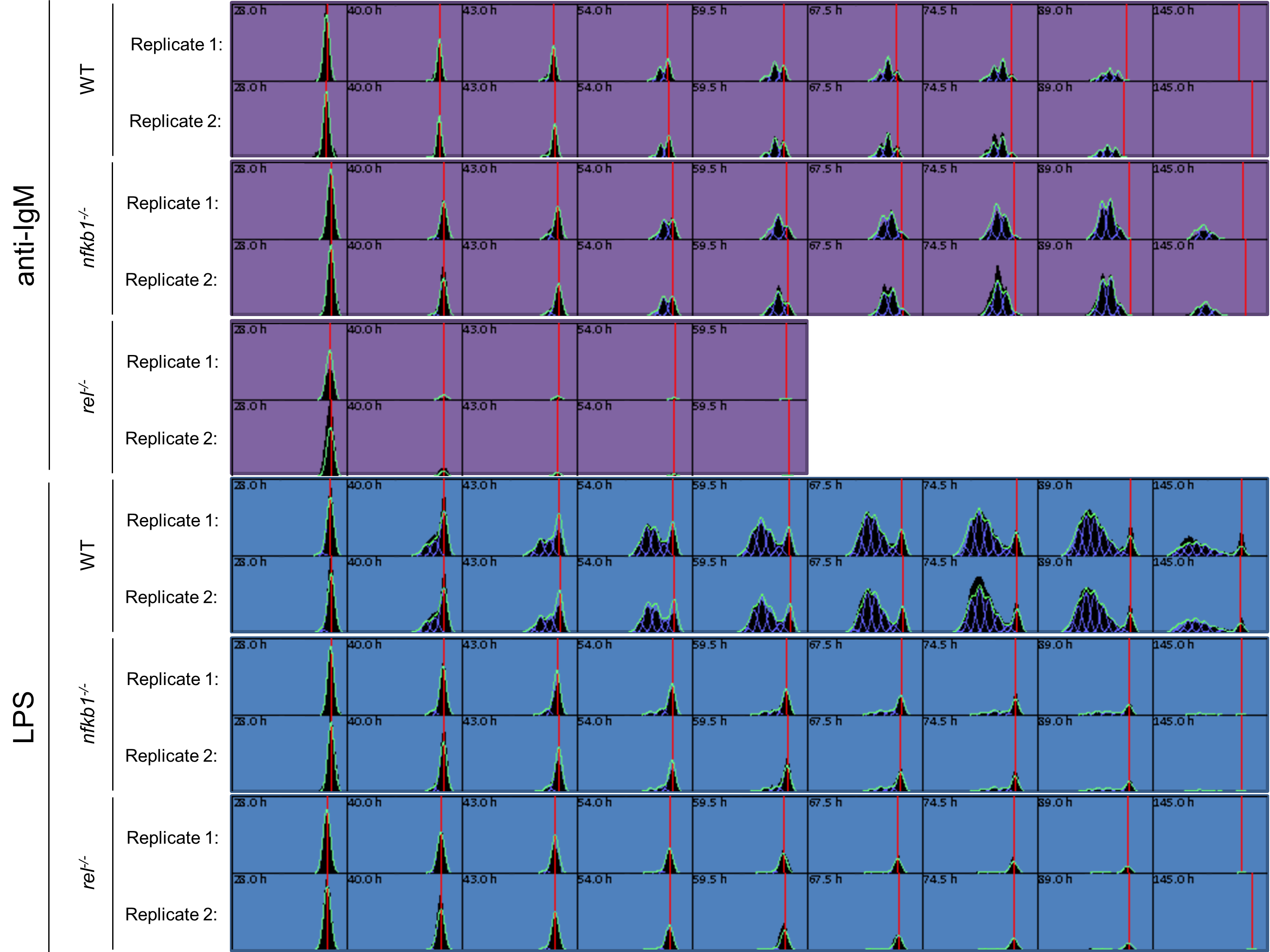

Supplement: Figure S6 — Best-fit fcyton solution overlays for stimulated wildtype, nfkb1−/− , and rel−/− B cell CFSE time courses. CFSE fluorescence data was collected and phenotyped using FlowMax, a computational tool that implements our integrated methodology. Green overlays show the weighted average best-fit model solutions for six duplicate log-fluorescence CFSE time courses (filled histograms). Columns represent individual time points. Histograms are normalized to the highest count for each time course across experimental duplicates. X-axes are in log-fluorescence units and automatically chosen to encompass all fluorescence values across all time-points and experimental runs. Red line shows manually selected position of the undivided population. Times of collection are indicated next to each histogram. Background indicates stimulus (blue = LPS, purple = anti-IgM). See also Figure 7. (TIF) [file pone.0067620.s006.tif]

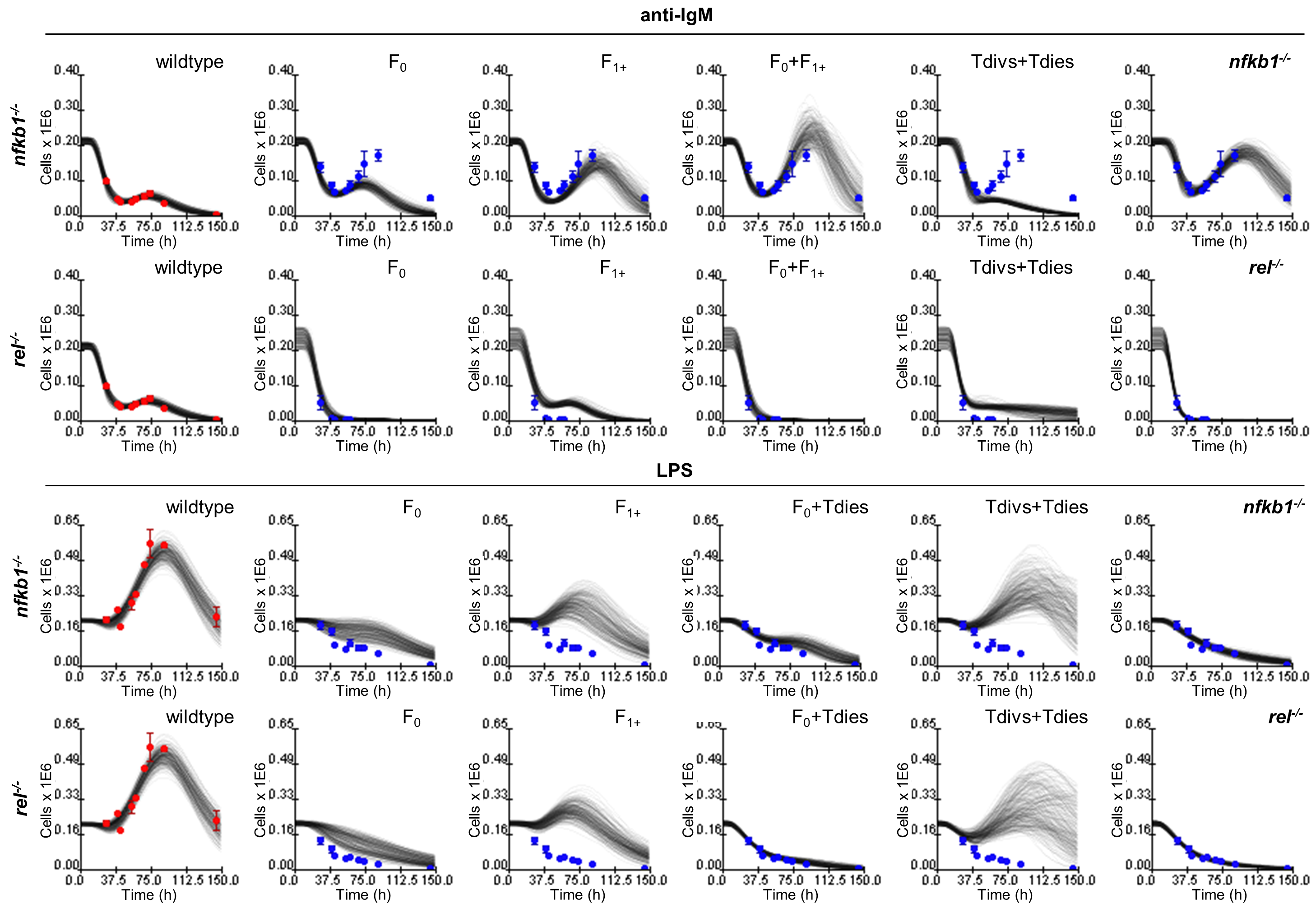

Supplement: Figure S7 — Using chimeric model solutions to identify key fcyton parameters. Total model cell counts determined when combinations of best-fit wildtype parameters were replaced by nfkb1 −/− -specific (rows 1 and 3) and rel−/−specific (rows 2 and 4) best-fit maximum-likelihood parameter ranges for anti-IgM (rows 1 and 2) and LPS (rows 3 and 4) stimulation. Dots show wildtype (red) and knockout (blue) experimental counts. Error bars show standard deviation of cell counts from duplicate runs. Poor fitting indicates that the indicated parameters do not sufficiently describe the mutant phenotype. (TIF) [file pone.0067620.s007.tif]
