# Supplementary material for: FlowMax: A Computational Tool for Maximum Likelihood Deconvolution of CFSE Time Courses
Source: PLoS One. 2013 Jun 27;8(6):e67620. doi: 10.1371/journal.pone.0067620 (PMC3694893; doi:10.1371/journal.pone.0067620)
Supplement: Table S1 — Analysis of fit running time dependence on the number of time points and generations. The average running time for fitting the cell fluorescence followed by fitting the fcyton cell population model using the best-fit cell fluorescence parameters to 300 generated time courses with four, seven, and ten time points is shown. Fitting was carried out using an assumed 6, 9, or 12 generations during fitting. Times are in minutes and errors are SEM. See also Table S3 and S4. (DOCX) [file pone.0067620.s008.docx]

| **Generations** | **Four Time Points** | **Seven Time Points** | **Ten Time Points** |
| --- | --- | --- | --- |
| 6 | 0.86±0.01 | 1.37±0.01 | 1.62±0.01 |
| 9 | 1.43±0.01 | 2.11±0.02 | 2.54±0.02 |
| 12 | 1.84±0.01 | 2.82±0.02 | 3.52±0.02 |
