# Supplementary material for: FlowMax: A Computational Tool for Maximum Likelihood Deconvolution of CFSE Time Courses
Source: PLoS One. 2013 Jun 27;8(6):e67620. doi: 10.1371/journal.pone.0067620 (PMC3694893; doi:10.1371/journal.pone.0067620)
Supplement: Table S2 — Starting and fitted cyton model parameters for four successful Cyton Calculator fitting trials. Starting cyton model parameter values that resulted in successful fits of our CFSE LPS-stimulated wildype B cell time course (columns 2–5) were chosen manually within ranges specified in Table S3. Corresponding Cyton Calculator [9] best-fit parameters are shown in columns 6–9. The data for experimental replicates is shown in Figure S6 (WT LPS). (DOCX) [file pone.0067620.s009.docx]

| **Parameter** | **Starting Value 1** | **Starting Value 2** | **Starting Value 3** | **Starting Value 4** | **Fitted Value 1** | **Fitted Value 2** | **Fitted Value 3** | **Fitted Value 4** |
| --- | --- | --- | --- | --- | --- | --- | --- | --- |
| **F_0_** | 0.001 | 1 | 0.57 | 0.72 | 0.41 | 0.67 | 0.47 | 0.37 |
| **E[Tdiv_0_]** | 30.6 | 80.8 | 41.2 | 33.8 | 39.3 | 45.1 | 43 | 38.6 |
| **s.d.[Tdiv_0_]** | 0.21 | 0.33 | 0.68 | 0.22 | 7.58 | 19.9 | 14.1 | 8.54 |
| **E[Tdiv_1+_]** | 17.6 | 49 | 20.7 | 14.5 | 14 | 17.7 | 9.43 | 8.96 |
| **s.d.[Tdiv_1+_]** | 0.18 | 0.35 | 0.36 | 0.35 | 13.3 | 11.6 | 0.48 | 2.2 |
| **E[Tdie_0_]** | 110 | 94.7 | 200 | 130 | 130 | 275 | 137 | 119 |
| **s.d.[Tdie_0_]** | 0.57 | 0.42 | 0.8 | 0.6 | 123 | 260 | 129 | 113 |
| **E[Tdie_1+_]** | 32.9 | 52.3 | 66.3 | 51.6 | 45.2 | 18.7 | 49.5 | 69.6 |
| **s.d.[Tdie_1+_]** | 0.8 | 0.5 | 0.17 | 0.8 | 42.7 | 0.95 | 46.8 | 47.2 |
| **D μ** | 1.04 | 1.16 | 2.37 | 4.7 | 3.16 | 1.37 | 1.57 | 1.23 |
| **D σ** | 1.28 | 0.53 | 4.91 | 5.7 | 1.53 | 5.9 | 1.95 | 1.95 |
