# Supplementary material for: FlowMax: A Computational Tool for Maximum Likelihood Deconvolution of CFSE Time Courses
Source: PLoS One. 2013 Jun 27;8(6):e67620. doi: 10.1371/journal.pone.0067620 (PMC3694893; doi:10.1371/journal.pone.0067620)
Supplement: Table S3 — Cell fluorescence and population parameter ranges used to generate realistic CFSE time courses. Selected ranges were chosen to exclude biologically implausible scenarios. Parameters were sampled evenly from the specified ranges whenever generating 1,000 time courses. The standard deviation parameters for the log-normal distributions: Tdiv0, Tdiv1+, Tdie0, Tdie1+ were further restricted to be less than or equal to their corresponding log-normal expected value parameters (e.g s.d[Tdiv0] ≤ E[Tdiv0]). Model fitting was restricted within these parameter ranges. Refer to Table S4 for the specific time points used. (DOCX) [file pone.0067620.s010.docx]

| **Cell Fluorescence Parameter** | **Minimum** | **Maximum** |
| --- | --- | --- |
| Peak width(CV) | 0.015 | 0.021 |
| Dye Dilution Ratio | 0.45 | 0.5 |
| Background Fluorescence | 0 | 1000 |
| Fluorescence Shift | -0.001 | 0.001 |
|  |  |  |
| **Population Parameter** | **Minimum** | **Maximum** |
| E[Tdiv_0_] | 6 h | 72 h |
| E[Tdie_0_] | 6 h | 192 h |
| E[Tdiv_1+_] | 6 h | 48 h |
| E[Tdie_1+_] | 6 h | 96 h |
| s.d.[Tdiv_0_] | 0.001 h | 72 h |
| s.d.[Tdie_0_] | 0.001 h | 192 h |
| s.d.[Tdiv_1+_] | 0.001 h | 48 h |
| s.d.[Tdie_1+_] | 0.001 h | 96 h |
| D μ | -3 generations | 8 generations |
| D σ | 0.001 generations | 10 generations |
| F_0_ | 0.001 | 1 |
| Start Cells (N) | 10,000 cells | 200,000 cells |
| K mech death | 0 | 0 |
| Fraction mech death | 0 | 0 |
