# Supplementary material for: FlowMax: A Computational Tool for Maximum Likelihood Deconvolution of CFSE Time Courses
Source: PLoS One. 2013 Jun 27;8(6):e67620. doi: 10.1371/journal.pone.0067620 (PMC3694893; doi:10.1371/journal.pone.0067620)
Supplement: Table S4 — Time points considered for analysis of generated time courses. For generated time courses, model solutions were sampled according to these time course schedules. Three, five, and ten time points were used in Figure S4. Four, four early, and eight time points were used in Figure 6. Four, seven, and ten time points were used when generating Table S1. Otherwise 10 time points were sampled from generated datasets. See also Table S3. (DOCX) [file pone.0067620.s011.docx]

| **Time Point** | **3 Time points** | **4 Time points** | **4 early time points** | **5 Time Points** | **7 Time Points** | **8 Time Points** | **10 Time Points** |
| --- | --- | --- | --- | --- | --- | --- | --- |
| 1 | 48 h | 24 h | 12 h | 24 h | 24 h | 24 h | 6 h |
| 2 | 96 h | 60 h | 24 h | 48 h | 48 h | 32 h | 24 h |
| 3 | 144 h | 96 h | 36 h | 72 h | 60 h | 48 h | 36 h |
| 4 |  | 144 h | 48 h | 96 h | 72 h | 60 h | 48 h |
| 5 |  |  |  | 144 h | 96 h | 80 h | 60 h |
| 6 |  |  |  |  | 144 h | 100 h | 72 h |
| 7 |  |  |  |  | 180 h | 144 h | 96 h |
| 8 |  |  |  |  |  | 192 h | 120 h |
| 9 |  |  |  |  |  |  | 144 h |
| 10 |  |  |  |  |  |  | 192 h |
