# Supplementary material for: FlowMax: A Computational Tool for Maximum Likelihood Deconvolution of CFSE Time Courses
Source: PLoS One. 2013 Jun 27;8(6):e67620. doi: 10.1371/journal.pone.0067620 (PMC3694893; doi:10.1371/journal.pone.0067620)
Supplement: Text S1 — Supplementary Methods. This text includes notes and method for: description of CFSE time courses, fitting the cell fluorescence model, peak weight calculations during cell fluorescence model fitting, fitting the fcyton model to cell counts derived from fluorescence histograms, fitting the fcyton models to fluorescence histograms directly, parameter sensitivity estimation, and clustering by sensitivity agglomeration. (DOC) [file pone.0067620.s012.doc]

**SUPPLEMENTARY METHODS**

**Description of CFSE time courses**

A typical CFSE time course consists of a set of time points. Each time point consists of one or more experimental bulk CFSE fluorescence measurements (experimental runs/replicates). Each experimental run consists of a set of CFSE fluorescence measurements which are typically represented by 2D log-transformed (or log-fluorescence) histograms. We assume that the total number of cells per run is also known.

**Fitting the cell fluorescence model**

The simulated annealing approach was used to arrive at the set of cell fluorescence parameters (CV, Ratio, Background, and Shift) that best describe a log-fluorescence histogram. Each simulated annealing optimization routine was initialized such that for time $t\in\{1,2,\ldots,n\}$:

| $CV_{t}=\left\{\begin{matrix} CV_{min} if t=1 \\ CV_{t-1} \end{matrix}\right.$, | (S1) |
| --- | --- |
| $Ratio_{t}=Ratio_{max}$, | (S2) |
| $Background_{t}=Background_{max}$, | (S3) |
| $Shift_{t}=0$, | (S4) |
| $CV_{min}= \left\{\begin{matrix} CV_{min} if t=1 \\ CV_{t-1}*(1-0.5^{t-1}) \end{matrix}\right.$, | (S5) |
| $CV_{max}= \left\{\begin{matrix} CV_{max} if t=1 \\ CV_{t-1}*(1+0.5^{t-1}) \end{matrix}\right.$. | (S6) |

where the subscripts “min” and “max” signify the given lower and upper limit for a parameter, respectively. We used the best-fit CV parameters from each time point to constrain the CV parameter range for the subsequent time point. This was useful for fitting later time points for which individual peaks were harder to discern without some prior knowledge of the approximate CV from previous time points.

In summary, if both early and late time points are available, FlowMax uses information from fitting the early time points to constrain the cell fluorescence parameters for later time points (described in the supplementary section “Fitting the cell fluorescence model”). In this way, if the CV or dye halving ratio “drifts” between time points, the early time points can be used to guide the fitting of these parameters for later time points. However, the position of the undivided population is one of the parameters supplied by the user for each histogram as it depends on the experimental conditions (cell staining conditions, cell type, dye catabolism, cytometer setup, etc.). Instead of trying to model how these factors affect the position of the undivided peak across time/generations (which is computationally intractable and outside of the scope of this study), we rely on the user to specify the approximate position of the undivided peak based on the location from previous time points. To help with this, we include a fourth “shift” term in the model to find the optimal peak placement.

A score-based annealing schedule was used during simulated annealing. Initial temperature was set to half of the fit score of the initial random parameter set. The minimum temperature was set to 1/1,000 of that value. Temperature was multiplied by 0.99 at the end of each iteration. In addition, if a better solution was found, the temperature was set to the minimum of the initial temperature or the current temperature multiplied by 1.1. This “heating” was used to discourage local trapping of the solution. In practice, this artificial heating helped in cases where solutions were stuck for many iterations in a high score parameter space but proceeded toward a better solution toward the end of the optimization, in which case, the sudden frequent improvement in the solution “reheated” the system and allowed for optimization to finish. While this approach resulted in satisfactory optimization, other optimization schemes of equal or better efficiency may be possible.

A scoring function which penalized both the squared distance as well as the difference in correlation between the fluorescence histograms for a particular time point, t, and experimental run, r

| $Score_{t,r}=\sqrt{\frac{\sum_{i=1}^{m} \left(n\cdot\left(H_{normal}\left[i\right]-M\left[i\right]\right)\right)^{2}}{m}}+n\cdot(1-cor\left(H_{normal},M\right))$, | (S7) |
| --- | --- |

was used where $H_{normal}$ is the histogram of d observed fluorescence values, M is the model histogram of fluorescence values, n is the number of cells measured experimentally, and cor(x,y) represents the Pearson correlation coefficient between the histograms of x and y. The average run score was used as the time point score, and the average time point score was used as the overall objective function during fitting. The scoring function was manually optimized for experimental data fitting: correlation was included to help guide initial optimization toward a suitable histogram shape.

While the choice of algorithm for fitting the cell-fluorescence model did not qualitatively affect the conclusions, we desired a fitting algorithm which would allow us to quickly find appropriate cell fluorescence model parameters (i.e. peak CV, ratio between peaks, autofluorescence, and offset from user-specified μ_0_), while taking into account solutions from previous time points. We also wanted to exclude solutions that were outside of user-specified ranges. In addition, we wanted a fitting approach which dealt with solution trapping in local minima, as is often the case with noisy experimental data. We are not aware of a conventional method for achieving this efficiently. Therefore, we selected the stochastic simulated annealing approach for fitting the model parameters, while explicitly solving for peak weights during each step (using a conventional non-linear regression approach described above), and allowing for information from earlier time points to contribute to the fit. Our evaluation of the fitting methodology suggests good agreement between fitted and generated data (see Fig. 2); however other methods for fitting may also be appropriate. In addition, our methodology can be adapted to incorporate other models for cell fluorescence and/or fitting approaches if desired by changing the cell fluorescence computational module.

**Peak weight calculations during cell fluorescence model fitting**

The problem of solving for the optimal model weights given the cell fluorescence parameters can be formulated as a nonlinear least squares regression problem. Given a log-fluorescence histogram with fluorescence, $x$, and bin size Δ$x$:

| $H(x)=\text{Pr}(x<X_{\text{i}}\leq x+\Delta x\vert X_{i}\in Data)$ | (S8) |
| --- | --- |

Defining the cellular fluorescence model solution, $F\left(x\right)$, as a weighted sum of Gaussian distributions,$G_{g}(x)$ :

| $F\left(x\right)=\sum_{g=1}^{G} w_{g}G_{g}(x)$ , | (S9) |
| --- | --- |

where w represents a weight multiplier and $G_{g}=G(\mu_{g},\sigma_{g})$. Then the sum of squared errors is:

| $E= \sum_{i=1}^{I} \left(H\left(i\right)-\int_{i-\Delta x}^{i} F\left(x\right)dx\right)^{2}$ . | (S10) |
| --- | --- |
| To solve for the optimal set of weights, minimize E. Rewriting E: |  |
| $E=\sum_{i=1}^{I} \left(H\left(i\right)-\int_{i-\Delta x}^{i} \sum_{g=1}^{G} w_{g}G_{g}\left(x\right)\text{ }dx\right)^{2}$ | (S11) |
| $=\sum_{i=1}^{I} \left(\sum_{g=1}^{G} w_{g}\int_{i-\Delta x}^{i} G_{g}\left(x\right)dx-H\left(i\right)\right)^{2}$ | (S12) |
| $=\sum_{i=1}^{I} \left(\sum_{g=1}^{G} w_{g}J_{ig}-H\left(i\right)\right)^{2}$. | (S13) |
| To solve the minimization problem, solve: |  |
| $0= \frac{\partial E}{\partial w_{b}}$ | (S14) |
| where:  $\frac{\partial w_{g}}{\partial w_{b}}=\delta_{gb}, \delta_{gb}=\left\{\begin{matrix} 1, g=b \\ 0, g\neq b \end{matrix}\right.$, | (S15) |
| $0=\sum_{i=1}^{I} 2\left(\sum_{g=1}^{G} w_{g}J_{ig}-H(i)\right)\left(\sum_{g=1}^{G} \delta_{gb}J_{ig}\right)$, | (S16) |
| $0=2\sum_{i=1}^{I} \left(\sum_{g=1}^{G} w_{g}J_{ig}-H(i)\right)\left(J_{ib}\right)$, | (S17) |
| $0=\sum_{g=1}^{G} w_{g}\left(\sum_{i=1}^{I} J_{ig}J_{ib}\right)-\sum_{i=1}^{I} H\left(i\right)J_{ib}$, | (S18) |
| $\sum_{g=1}^{G} \left(\sum_{i=1}^{I} J_{ig}J_{ib}\right)w_{g}=\sum_{i=1}^{I} H\left(i\right)J_{ib}$, | (S19) |
| $M\vec{w}=\vec{c}$. | (S20) |

*M* is a *G x G* matrix and both $\vec{c}$ and $\vec{w}$are vectors of size G, where G is the number of generations being modeled. To calculate each element of M, and $\vec{c}$:

| $M_{ba}= \sum_{i=1}^{I} \int_{i-\Delta x}^{i} G_{a}\left(x\right)dx\int_{i-\Delta x}^{i} G_{b}\left(x\right)dx$, | (S21) |
| --- | --- |
| $c_{j}= \sum_{i=1}^{I} H(i)*\int_{i-\Delta x}^{i} G_{j}\left(x\right)dx$, | (S22) |

where $G_{x}$ is the Gaussian distribution $G_{x}(_{x},_{x})$ representing fluorescence distributions for cells in division class x from equations (1) and (2). Solving for the optimal weights,$\vec{w},$ associated with each Gaussian distribution:

| $\text{ }\vec{w}=M^{-1}\vec{c}$, | (S23) |
| --- | --- |

we further ensure that the weights are non-negative and sum to 1:

| $w_{g}>0 \forall w_{g}\in\vec{w}$,  $\sum_{g=1}^{G} w_{g}=1$. | (S24)  (S25) |
| --- | --- |

By solving for the weights at the end of each iteration of fluorescence model optimization (see above), excellent fluorescence model parameters were found relatively quickly. Furthermore, solving explicitly for the peak weights subjected only the cell fluorescence model parameters to optimization, minimizing fitting bias that could arise due to errors in weight optimization.

**Fitting the fcyton model to cell counts derived from fluorescence histograms**

In order to fit the fcyton cell population model parameters ($N$, $F_{0}$, $D\mu$, $D\sigma$, $Tdiv_{0}\mu$, $Tdiv_{0}\sigma$, $Tdie_{0}\mu$, $Tdie_{0}\sigma$, $Tdiv_{1+}\mu$, $Tdiv_{1+}\sigma$, $Tdie_{1+}\mu$, $Tdie_{1+}\sigma$) to sets of division specific cell counts, a simulated annealing approach was used. Specifically, fitting started with 4,000 iterations of randomized guess and check trials to establish a coarse approximation of the parameters. Then the best parameter set out of the 4,000 randomly selected parameter sets was used as the starting point for simulated annealing. Prior to the first round of optimization by simulated annealing, the temperature, T, was set to the current best-fit score, and T_min_, or the stopping temperature, was initialized to 1/100,000 of T. Each simulated annealing iteration consisted of three steps: the current parameters were modified slightly (Gaussian sampling with sigma = 1% of the current value), the fit score was calculated for the new parameter set, and the new parameter set was adopted with probability p such that:

| $p=\left\{\begin{matrix} 1, if the new score was lower \\ e^{-\frac{diff\cdot k}{T}}, otherwise \end{matrix}\right.$. | (S26) |
| --- | --- |

where *diff* is the new score minus the previous score and k is a score scaling factor.

Similarly to the optimization process used to fit the fluorescence model, if the new score was an improvement over the current global best fit score, the temperature was set to the minimum of the current temperature*1.1 or the initial temperature. This artificial “heating” was implemented to ensure that all improving parameter sets find a local minimum after leaving a parameter space of relatively poor fit scores. The annealing factor was 0.9995.

Two objective functions were used during this optimization process: a simple objective function consisting of the square deviation between the model and generated counts, and a more complex optimized objective function that takes into account multiple difference measures:

| $Obj_{SD}=\sum_{t=1}^{n} \sum_{i=1}^{G} \left(Count_{data}\left[t\right]*Prop_{data}\left[i,t\right]-Count_{model}\left[t\right]*Prop_{model}\left[i,t\right]\right)^{2}$ | (S27) |
| --- | --- |
| $Obj_{OPT}=\left\{\begin{matrix} \sum_{t=1}^{n} \left[\begin{matrix} 2*\frac{cor\left(Prop_{data}\left[t\right],Prop_{model}\left[t\right]\right)}{n}+ \\ \left(\frac{Count_{data}\left[t\right]}{Count_{data}\left[t\right]+Count_{model}\left[t\right]}-\frac{Count_{model}\left[t\right]}{Count_{data}\left[t\right]+Count_{model}\left[t\right]}\right)^{2}+ \\ 5*\sum_{i=1}^{G} \frac{\left(Prop_{data}\left[i,t\right]-Prop_{model}\left[i,t\right]\right)^{2}}{n} \end{matrix}\right]+ \\ 2*cor(Count_{data},Count_{model}) \end{matrix}\right\}$ | (S28) |

where *Count*[t] represents the total cell count at time point t, and *Prop*[i,t] is the proportion of cells at time point t in generation i. The cor(x,y) function represents the Pearson correlation coefficient between the functions x and y. In the optimized scoring function, G is defined as the maximum number of division classes at time point t such that *Count_data_*[G+1] = 0 and G > 6. Therefore, all cells in division classes > 6 are treated as one population in order to reduce the penalty for cells assigned to division classes 7 and higher. This was implemented due to an increased difficulty in discriminating CFSE peaks after 6 divisions experimentally. This complex objective function was developed in a step-by-step manner, starting with a basic sum of squared differences objective function (S27), followed by the addition of correlations, and the use of *ad* hoc weights for each part of the scoring function. The annealing schedule was optimized to allow for efficient optimization of both fit scores.

After an initial round of simulated annealing, a subsequent fast round of localized simulated annealing centered on the optimal solution was carried out. Analogously to the first round, the temperature was set equal to the fit score for the current optimal solution and annealing continued until the temperature dropped below 1/1,000 of this temperature. At the beginning of each iteration a new set of parameters was generated randomly by modifying the best parameter set (Gaussian sampling with sigma = 0.1% of the current value). This limited the search to the local parameter space around the best-fit solution. In practice this often only yielded minor improvements to the fit, but on rare occasions, allowed a very poor solution to be improved significantly before it was used in the post-processing steps following model parameterization.

**Fitting the fcyton models to fluorescence histograms directly**

Analogously to the fitting approach used to fit the fcyton models to generational cell counts, a stochastic method was used to fit the models to experimental fluorescence histograms directly. The fluorescence model was used to adapt the derived model cell counts to log-fluorescence histograms. Specifically, for each set of predicted division-specific cell counts a fluorescence histogram was generated using the previously fitted cell fluorescence parameters and generational cell counts as weights (see Fig. 2a). This facilitated the use of a relatively simple objective function for calculating the difference between the model fluorescence histograms and the experimental fluorescence histograms. A manually optimized objective function which accounted for the difference between the fluorescence histograms, the correlation between the fluorescence histograms, and the proportion of the total cells represented in each histogram was used:

| $Obj_{MRSD+}=\frac{\sum_{i=1}^{t} \sum_{j=1}^{r} Cells_{i,j}\cdot\frac{\sqrt{\sum_{k=1}^{m} \left(H_{i,j}\left[k\right]-M_{i}\left[k\right]\right)^{2}}}{cor\left(H_{i,j},M_{i}\right)^{2}}}{\sum_{i=1}^{t} \sum_{j=1}^{r} Cells_{i,j}}$. | (S29) |
| --- | --- |

where $Cells_{i}^{j}$ is the total cell count in run j for time point i, and cor(x,y) represents the Pearson correlation coefficient between the experimental histogram, $H_{i}^{j}$, and modeled histogram, $M_{i}$ . We also tested if simpler scoring functions were sufficient for fitting generated datasets. Specifically we tried a simple mean absolute deviation objective function (MAD):

| $Obj_{MAD}=\frac{\sum_{i=1}^{t} \sum_{j=1}^{r} Cells_{i,j}\cdot\sum_{k=1}^{m} \left\vert H_{i,j}\left[k\right]-M_{i}\left[k\right]\right\vert}{\sum_{i=1}^{t} \sum_{j=1}^{r} Cells_{i,j}}$, | (S30) |
| --- | --- |

as well as a mean root square deviation objective function:

| $Obj_{MRSD}=\frac{\sum_{i=1}^{t} \sum_{j=1}^{r} Cells_{i,j}\cdot\sqrt{\sum_{k=1}^{m} \left(H_{i,j}\left[k\right]-M_{i}\left[k\right]\right)^{2}}}{\sum_{i=1}^{t} \sum_{j=1}^{r} Cells_{i,j}}$, | (S31) |
| --- | --- |

While even the simplest scoring function we tried produced similar results, the more complex objective function produced lower average errors in generational counts and outperformed the simplest (MAD) objective function when fitting the fcyton N parameter (see Figure S5 and main text). Therefore, we decided that the more complex objective function (MRSD+) was justified.

**Parameter sensitivity estimation (cont.)**

A simple divide-and-conquer algorithm was chosen for calculating the parameter sensitivity ranges which operates on the best-fit fcyton model parameters, X = {$N$, $F_{0}$, $D\mu$, $D\sigma$, $Tdiv_{0}\mu$, $Tdiv_{0}\sigma$, $Tdie_{0}\mu$, $Tdie_{0}\sigma$, $Tdiv_{1+}\mu$, $Tdiv_{1+}\sigma$, $Tdie_{1+}\mu$, $Tdie_{1+}\sigma$}, and a constant values for the fit tolerance,$\varepsilon$ :

ALGORITHM CalParamSensitivities (*X*, ε)

$$MinScore\leftarrow AreaDiff(X)$$

Define array Upper of size |X|

Define array Lower of size |X|

FOREACH parameter:

$$Original=x_{i}$$

$$StepSize\leftarrow\frac{|x_{i}-\max(x_{i})|}{1000}$$

WHILE $StepSize>\frac{|x_{i}-\max(x_{i})|}{10000}$

$$x_{i}\leftarrow x_{i}+StepSize$$

$$CurrScore\leftarrow AreaDiff(X)$$

IF $CurrScore<MinScore\cdot(1+\varepsilon)$

$$x_{i}\leftarrow x_{i}-StepSize$$

$$StepSize\leftarrow\frac{StepSize}{2}$$

ELSE

$$StepSize\leftarrow\min(StepSize\cdot2,\max(x_{i})-x_{i})$$

ENDIF

ENDWHILE

$$Upper_{i}\leftarrow x_{i}$$

$$x_{i}=Original$$

ENDFOREACH

FOR EACH parameter:

$$Original=x_{i}$$

$$StepSize\leftarrow\frac{|\min(x_{i})-x_{i}|}{1000}$$

WHILE $StepSize>\frac{|\min(x_{i})-x_{i}|}{10000}$

$$x_{i}\leftarrow x_{i}-StepSize$$

$$CurrScore\leftarrow AreaDiff(X)$$

IF $CurrScore<MinScore\cdot(1+\varepsilon)$

$$x_{i}\leftarrow x_{i}+StepSize$$

$$StepSize\leftarrow\frac{StepSize}{2}$$

ELSE

$$StepSize\leftarrow\min(StepSize\cdot2,x_{i}-\min(x_{i}))$$

ENDIF

ENDWHILE

$$Lower_{i}\leftarrow x_{i}$$

$$x_{i}=Original$$

ENDFOREACH

RETURN Upper and Lower

Since the value of the tolerance parameter ε, or the maximum percent increase in the overall normalized percent area error (NPAE), is an important implicit parameter for sensitivity analysis, an appropriate value must be used. In our experience, a value that is too small resulted in small sensitivity ranges for each parameter tested and a large number of non-overlapping solutions. Conversely, high tolerance resulted in large acceptable parameter sensitivity ranges, increasing the chance that linear sampling of parameters introduced errors into sensitivity estimation and underestimating the sensitivity of the solution to model parameters. The parameter ε was set to 1% normalized percent area error (NPAE) empirically because this resulted in parameter sensitivities of approximately ±10% of the best-fit parameter value for the determinable parameters such as F_0_, Tdiv_0_, and Tdiv_1+_. In addition, this resulted in fewer than three solution clusters for experimental datasets. Conversely, values of ε smaller than 1% resulted in more than five solution clusters for the experimental datasets.

**Clustering by sensitivity agglomeration (cont.)**

The main motivation for clustering parameters is to identify solution uniqueness since more than one set of model parameters can sufficiently describe the data. To determine if more than one solution space can fit the data, we perform a parameter sensitivity analysis for each best-fit solution then successively combine solutions with the largest overlap in the solution space until only non-disjoint solutions remain. By doing this we guarantee that the clustered solution space(s) are non-redundant.

Since parameter fits are obtained using a non-deterministic fitting procedure, the best-fit parameter solution is only an estimate of the true solution. Furthermore, we established that parameters exhibit widely varying degrees of sensitivity that is heavily determined by the underlying dataset. Therefore, we first estimate the sensitivity of each best-fit parameter in a solution, obtaining a set of independent lower and higher parameter values for each parameter, p. Repeating the fitting n times we get the set of best-fit parameter ranges for parameter p:

| . $S_{p}=\left\{[L_{p,1},H_{p,1}],[L_{p,2},H_{p,2}],\ldots,[L_{p,n},H_{p,n}]\right\}.$ | (S32) |
| --- | --- |

We assume that each distribution of p found is equally likely and therefore the probability of p is modeled as:

|  | (S33) |
| --- | --- |

Where $I(L_{p,i}\leq x\leq H_{p,i})$is the indicator function and returns 1 if $p$ is within the i^th^ range. In other words, assumes a linear combination of uniform distributions for $p$, which comes about if one marginalizes the distribution on all other model parameters. Since represents the probability distribution model of the parameter p given the observed best-fit ranges, the region of maximum likelihood is the range of parameter values such that the overlap count is maximized. Furthermore, by ensuring that only overlapping parameter ranges are clustered together, we guarantee that only one such maximum region exists for each cluster:

|  | (S34) |
| --- | --- |

The maximum likelihood estimator for $p$ are all values of $p$ within the range $[\mathop{L_{p}}\limits^{\wedge} ,\mathop{H_{p}}\limits^{\wedge} ]$ or the intersection of all of the parameter ranges in $S_{p}$. In other words, $\mathop{L_{p}}\limits^{\wedge}$ and $\mathop{H_{p}}\limits^{\wedge}$are the maximum likelihood estimators of the lower and upper value of $p$, due to the resolution of the data and methodology (i.e. the probability of $p$ is uniform for any i^th^ best-fit sensitivity range $[L_{p,i},H_{p,i}]$). We report the region of$p$defined by $\mathop{L_{p}}\limits^{\wedge}$ and $\mathop{H_{p}}\limits^{\wedge}$for which is maximized, or the mode of .

An added potential benefit of clustering is to minimize bias in how we calculate the sensitivity of each solution. Since solution spaces are created by varying parameters around the best-fit value, an implicit parameter is the amount by which a solution can worsen as we vary each parameter independently. If the allowed error is inappropriate, the clustering will either return too many similar solutions (error tolerance too low) or both clusters with poor and good fits (tolerance is too high). The later happens because only the intersection of parameter spaces is kept during the agglomeration process, which means that an overestimate of the parameter range for solutions about a region containing a local minimum will still only contain good fits, while an over estimate of the parameter range for solutions about a region containing poor fits will contain poor solutions. A subsequent filtering of the solution clusters can be used to remove these “artifact” clusters containing poor solutions and we can alleviate the bias in selecting the error tolerance parameter.

Choosing the representative parameter value (the cluster “average”) for a specific cluster is somewhat arbitrary as the probability of the parameter within a range was assumed to be uniform during clustering. In reality, the probability of any parameter $p$ is likely to decrease with the distance from a best-fit value. Therefore when clustering parameter ranges, we keep track of a weighted average value of $p$ that is guaranteed to be within the overlap between ranges being clustered, but its position is weighted according to the relative maximum distance from the average of each of the starting cluster averages:

| $\begin{matrix} d_{a}=H_{c}-A_{a}, \\ d_{b}=A_{b}-L_{c}, \\ A_{c}=\frac{d_{b}}{d_{a}+d_{b}}\left[H_{c}-L_{c}\right]+L_{c}=H_{c}-\frac{d_{a}}{d_{a}+d_{b}}\left[H_{c}-L_{c}\right], \end{matrix}$ | (S35) |
| --- | --- |

where the distance ($d$), high($H$), average ($A$), and low ($L$) values are used to agglomerate clusters a and b into cluster c and letting$A_{a}$<$A_{b}$.

The pseudo code for clustering the candidates by maximizing the parameter sensitivity overlap given the set of candidate best-fit fcyton parameter values and their corresponding lower and upper bound sensitivity ranges is shown below:

ALGORITHM: *ClusterBySensitivityAgglomeration (Candidates, Sensitivities)*

Define $Clusters=\{C_{1}=\left\{S_{1}\right\},C_{2}=\left\{S_{2}\right\}, \ldots,C_{n}= \left\{S_{n}\right\}\}$

Define $Averages=\{A_{1}=S_{1},A_{2}=S_{2}, \ldots, A_{n}=S_{n}\}$

Define $Lows=\{D_{1}=L_{1},D_{2}=L_{2}, \ldots,D_{n}= L_{n}\}$

Define $Highs=\{U_{1}=H_{1},U_{2}=H_{2}, \ldots, U_{n}=H_{n}\}$

Define *best_c1* = *best_c2* = -1

Define *largestOverlap* =0

Define *clustering* = true

WHILE *clustering* = true

FOR $A_{a}\in Averages, A_{b}\in Averages, a\neq b$

Define *dist* = 0

FOREACH parameter, *i*

Define $d_{i}=\left\{\begin{matrix} \frac{\left[\left(A_{b}\left[i\right]+U_{b}\left[i\right]\right)-(A_{a}\left[i\right]-D_{a}[i])\right]}{|A_{a}\left[i\right]+U_{a}[i]|+|A_{b}[i]+U_{b}[i]|}, if A_{a}>A_{b} \\ \frac{\left[\left(A_{a}\left[i\right]+U_{a}\left[i\right]\right)-(A_{b}\left[i\right]-D_{b}[i])\right]}{|A_{a}\left[i\right]+U_{a}[i]|+|A_{b}[i]+U_{b}[i]|}, if A_{a}\leq A_{b} \end{matrix}\right.$

d*ist = dist+d_i_*

ENDFOREACH

IF *dist* > 0 and *dist > largestOverlap*

*largestOverlap =dist*

*best_c1* = ­*a*

*best_c2* = b

ENDIF

ENDFOR

IF *best_c1* ≠ -1 and *best_c2­* ≠ -1

Define $newA,newD,newU$

FOREACH parameter, *i*

Define x = y=z=0

IF $A_{best\_c1}[i]$ <$A_{best\_c2}[i]$

$$x=D_{best\_c2}[i]$$

$$y=U_{best\_c1}[i]$$

$$z=A_{best\_c2}[i]-A_{best\_c1}[i]$$

ELSE

$$x=D_{best\_c1}[i]$$

$$y=U_{best\_c2}[i]$$

$$z=A_{best\_c1}[i]-A_{best\_c2}[i]$$

ENDIF

Define $x_{\mathrm{int}}=\frac{z\cdot y}{y+x}$

$$newA\left[i\right]=$$

$$A_{best\_c1}[i]+x_{\mathrm{int}}$$

$$newD[i]=$$

$$newA[i]-\max(A _{best\_c1}[i]-L_{best\_c1}[i],A _{best\_c2}[i]-L_{best\_c2}[i])$$

$$newU[i]=\min(A_{best\_c1}[i]+U_{best\_c1}[i],A_{best\_c2}[i]+U_{best\_c2}[i])-newA[i]$$

ENDFOREACH

$Averages =(Averages/\{\text{A}_{\text{best}_{\text{c}1}},A_{best_{c2}}\}$)∪$newA$

$Lows =(Lows$*/*{$D_{best\_c1}$,$D_{best\_c2}$})∪$newD$

$Highs =(Highs$*/*{$U_{best\_c1}$,$U_{best\_c2}$})∪$newU$

$Clusters =(Clusters$*/*{$C_{best\_c1}$,$C_{best\_c2}$})∪$(C_{best\_c1}C_{best\_c2}$)

ELSE

*clustering* = false

ENDIF

ENDWHILE

RETURN <$Averages,Lows,Highs,Clusters$>

END

**References**

1. S K, D GC, P VM (1983) Optimization by Simulated Annealing. Science New Series: 671-680.

2. Banks HT, Sutton KL, Thompson WC, Bocharov G, Doumic M, et al. (2011) A new model for the estimation of cell proliferation dynamics using CFSE data. J Immunol Methods 373: 143-160.

3. Luzyanina T, Roose D, Schenkel T, Sester M, Ehl S, et al. (2007) Numerical modelling of label-structured cell population growth using CFSE distribution data. Theor Biol Med Model 4: 26.
