# Supplementary material for: FlowMax: A Computational Tool for Maximum Likelihood Deconvolution of CFSE Time Courses
Source: PLoS One. 2013 Jun 27;8(6):e67620. doi: 10.1371/journal.pone.0067620 (PMC3694893; doi:10.1371/journal.pone.0067620)
Supplement: Text S2 — Succinct FlowMax tutorial. This text describes the typical steps required to build CFSE log-fluorescence histograms from raw fcs datasets, apply the integrated fitting methodology, and interpret the results. (DOC) [file pone.0067620.s013.doc]

**SUCCINCT FlowMax TUTORIAL**

Using FlowMax to phenotype CFSE time courses in terms of cellular parameters involves four parts:

1. Installation
2. Pre-processing
   1. Fluorescence data import
   2. Gate construction
   3. 1D log fluorescence histogram construction and selection for phenotyping
3. Model fitting
   1. Selection of appropriate time course
   2. Setting advanced options for phenotyping
4. Data visualization and post-processing
   1. Appropriate solution cluster selection
   2. Solution visualization.
   3. Determining if two solutions are different
   4. Data presentation/analysis

**Installation**

FlowMax was designed to work as a standalone cross-platform analysis tool. Therefore, there is only one file, FlowMax.jar, which can be launched using a Java 1.6 compatible Java Virtual Machine. Please make sure that FlowMax is granted the appropriate permissions to create new sub-folders (Launch in a non-restricted folder or as administrator/root). FlowMax will create a “Phenotypes” subfolder for storing phenotyping results. Note, since FlowMax workspace files do not store all of the FCS data, please ensure that the necessary FCS files are located in the same relative location to the workspace file (*.wrk).

**Pre-processing**

After carrying out a dye dilution time course (CFSE time course), export all of the flow cytometry data as FCS3.0 files. Launch FlowMax. If a workspace was saved from an earlier time, it can be accessed with the “Load Workspace” button. When FlowMax is first launched an empty workspace with some basic reminders is displayed in the center panel:

**
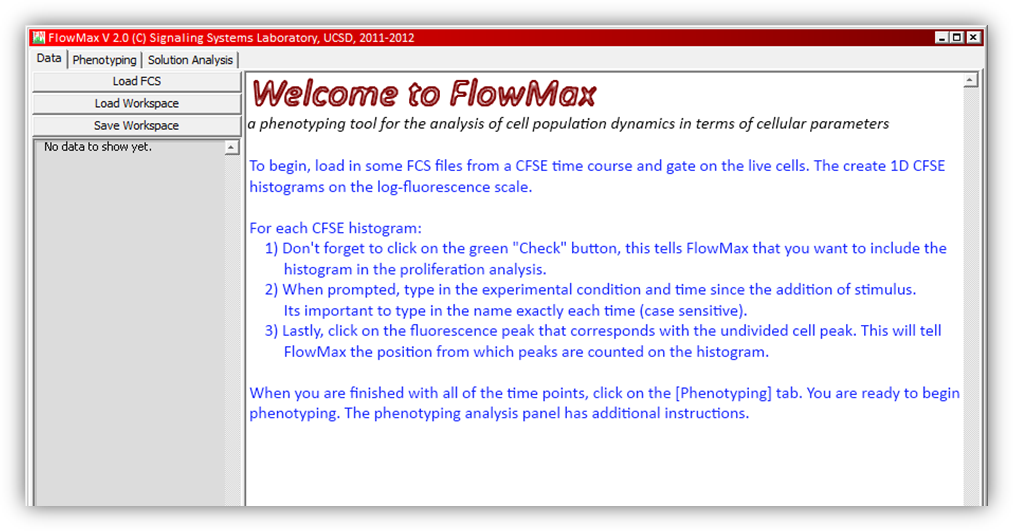
**

See troubleshooting tips if you are having trouble loading a saved workspace.


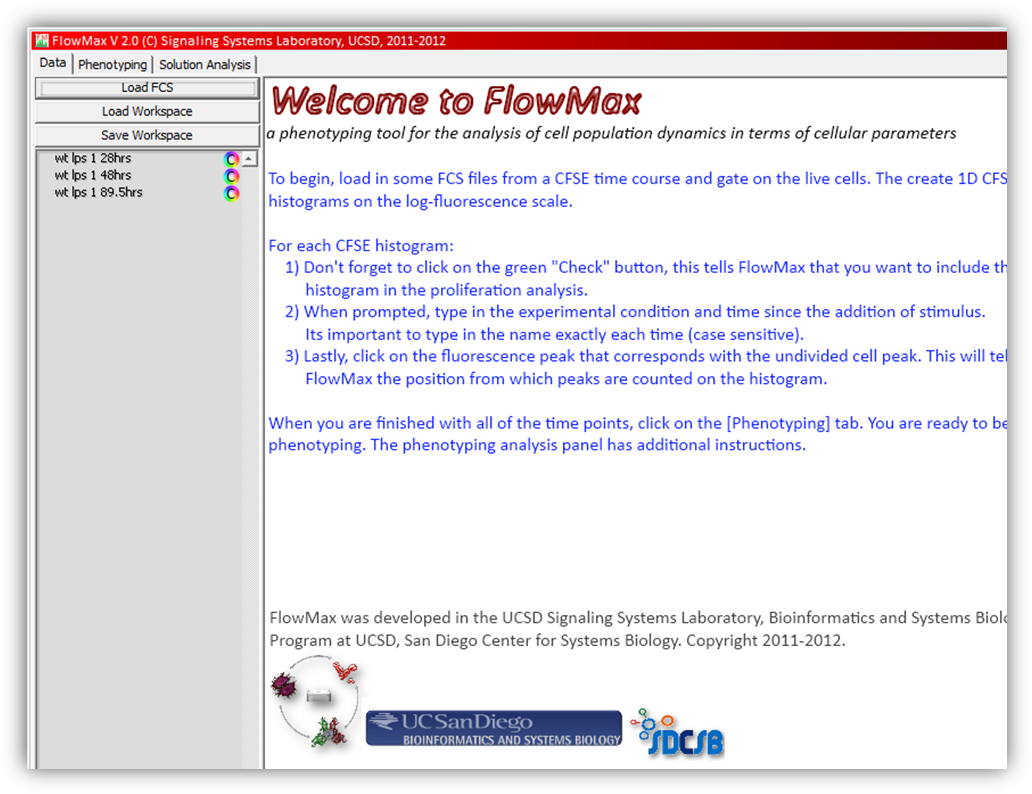
Load the FCS files into the workspace by clicking the “Load FCS” button at top left and selecting all FCS files in the dialog.


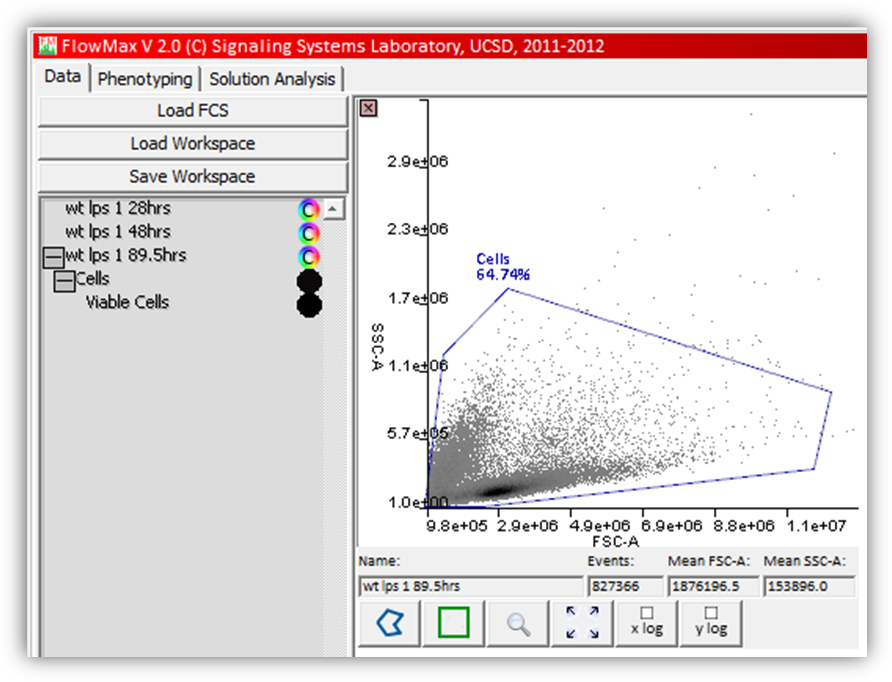
Now, double-click the first dataset, and create gates for viable cells of interest:

Polygon gate tool

Rectangular gate tool

Magnifying Lens Tool

Zoom Out

Change between linear/log axes

Basic statistics linear/log axes

Switch the x and y axes by right/ctrl clicking on the x and y axis labels. The default values are the first and second fluorescence parameter recorded by the flow cytometer (forward and side scatter in this example). Software compensation between parameters is possible by clicking on the rainbow circle at the right of each dataset listed. Gated data updates automatically.


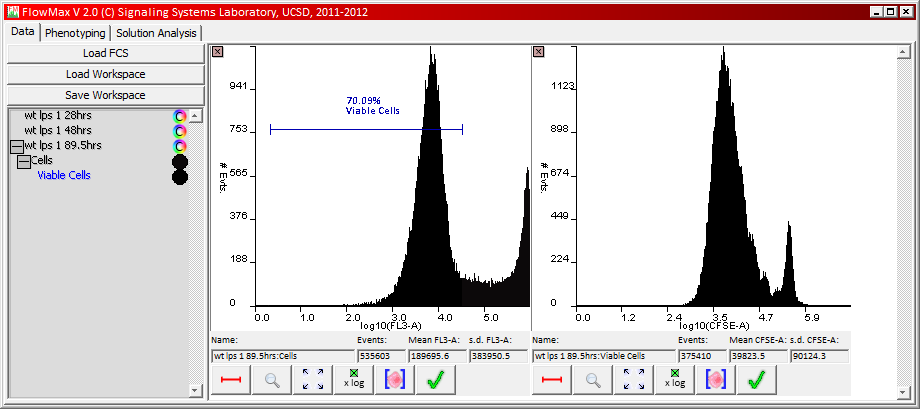
After non-viable cells are gated out, create a log-fluorescence histogram for the division tracking dye used. In this example, we used CFSE fluorescence as a marker for cell division:

Now gates can be copied to the other time points by first selecting the gate, then either pressing Ctrl+C to copy or right-clicking and selecting “copy gate”, and finally selecting a different gate/dataset and pressing Ctrl+V or right-clicking and selecting “paste gate”:


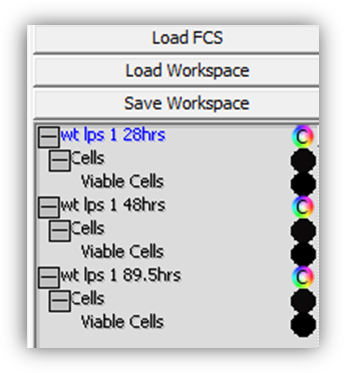


Click on the CFSE log-fluorescence histograms that were created for each time point and click the green “check” button that appears under the histogram statistics. A popup box will prompt for the experimental condition the time point should be tied to (keep these consistent for each time point), the next dialog requires the time after stimulus that corresponds with the current time point (in hours). Next, click on the generation 0 peak (undivided peak). A red line should appear indicating the peak that was selected:


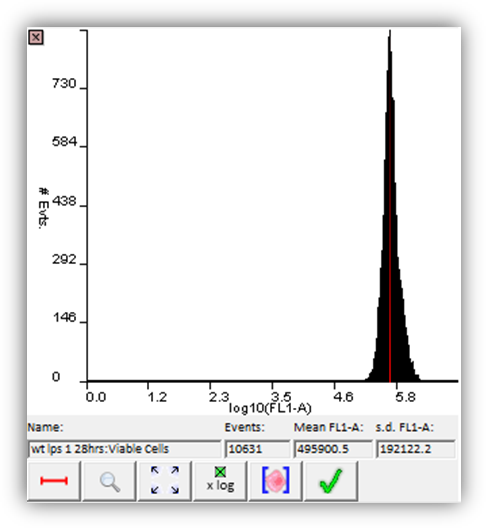

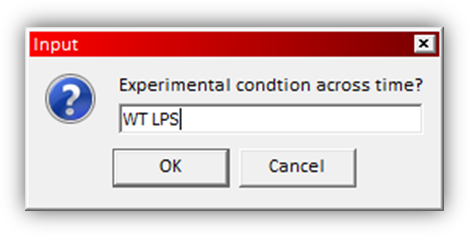


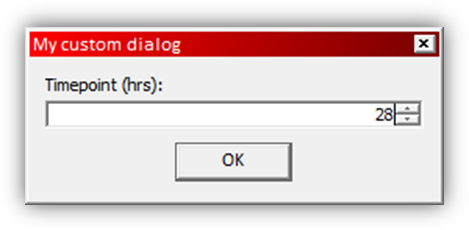


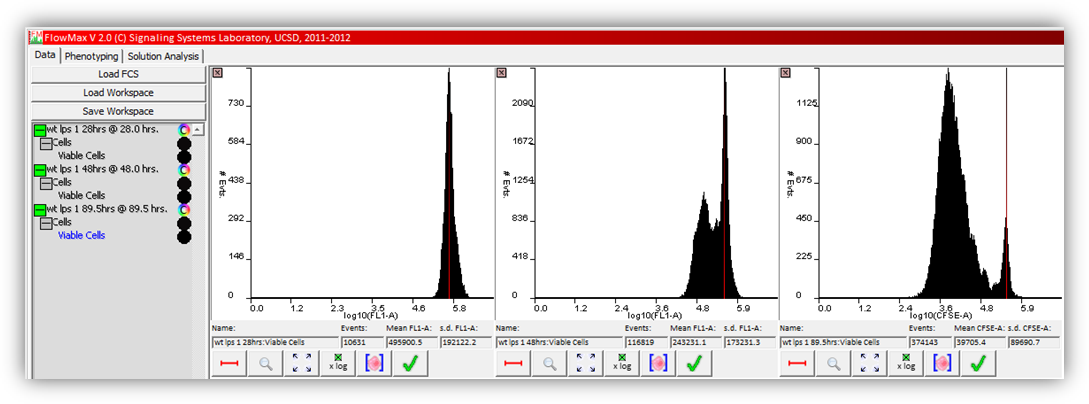
The box next to the file loaded will become green, indicating that the dataset has been prepared for phenotyping. Repeat this process for all time points:

**Model Fitting**


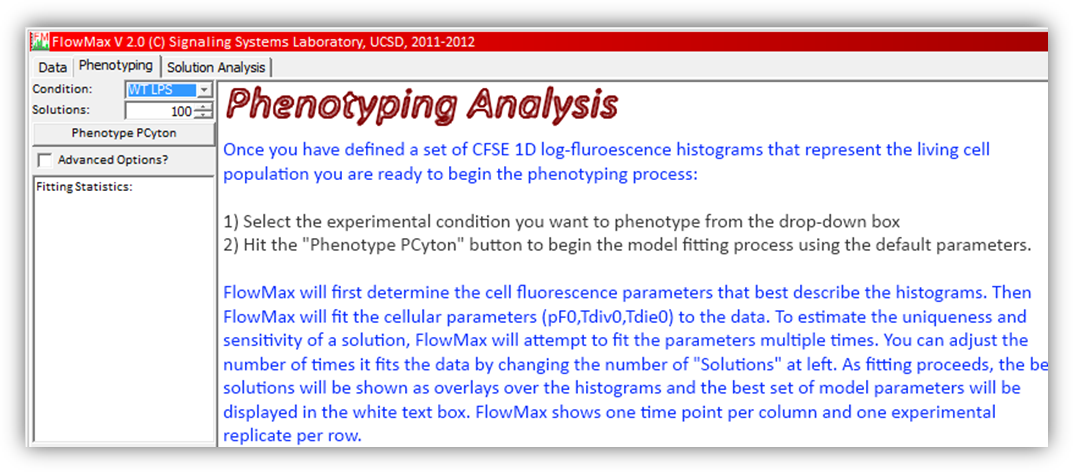
Now switch tabs to the “Proliferation” view and select the experimental condition for phenotyping:


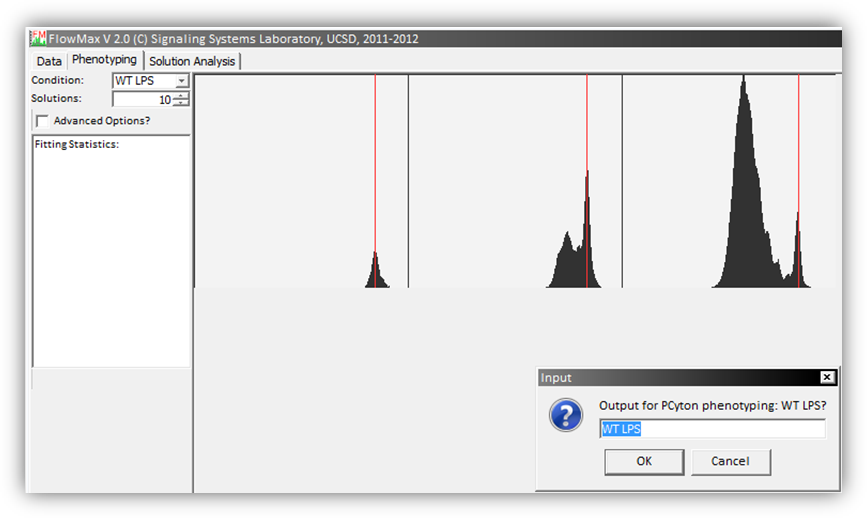
Adjust any of the “advanced” options from the advanced options panel if needed (such as the allowable ranges for the cell fluorescence and population parameters during fitting), and hit the “phenotype Pcyton” button. FlowMax will now load all the log-fluorescence histograms for the selected experimental condition (time course), and prompt for the phenotype name:

During fitting, FlowMax will first fit the cell fluorescence model to determine the best-fit cell fluorescence parameters, and then subsequently use those parameters to fit the best-fit cell population parameters (Tdiv_0_, pF_0_, Tdie_0_, etc.) from the histogram data. Furthermore, to estimate the sensitivity and uniqueness of the phenotyping solutions, FlowMax will repeat the pcyton fitting procedure multiple times and cluster the results (see the main text and methods for details). The number of solutions, the allowed ranges for parameters, and the number of generations to model are adjustable from the main panel (Advanced Options). Alternatively, the user can use the default parameter ranges, generations to model, and fit count. The estimate of solution uniqueness and parameter sensitivity can only improve with more solutions. Increasing the number of solutions will improve chances for a more accurate fit and may also help constrain solution parameter ranges after clustering.

**Data visualization and post-processing**

After phenotyping is complete, FlowMax will calculate the parameter sensitivity of each solution, filter out solutions that are worse than 0.1% of the best solution (NPAE see above), and cluster the solutions to produce one or more non-overlapping sets of cellular parameters along with their corresponding sensitivity ranges. As mentioned above, due to the clustering method used, each range of parameter values will represent the weighted shared sensitivity for all solutions that are part of the cluster (see above).

After phenotyping is finished, FlowMax will save all of the results under the Phenotypes/Exp subfolder, where Exp is the name provided at the start of the phenotyping.

Best-fit solution clusters can be visualized and further analyzed from the “Solution Analysis” tab:

**
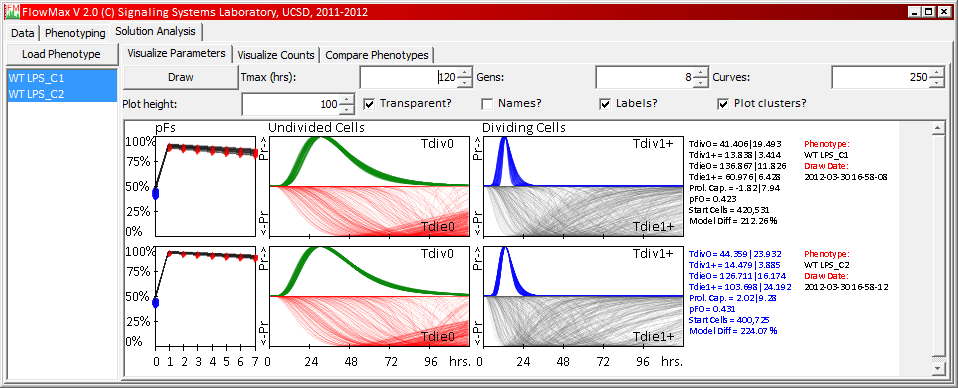
**

You can visualize the parameter distributions, the generational and total cell counts, compute the probability that one solution is different from another for a given log-fluorescence time course, and generate hybrid solutions using parameters from two different solutions (this can be used to qualitatively determine if a certain combination of parameters is necessary and/or sufficient to account for biological differences of two experiments. Images can be copied to the clipboard by right-clicking.

Furthermore, the full model solutions are saved in the form of comma-separated Excel files (*.csv). The results are saved to the directory that you specified at the start of the modeling fitting step. Open up the csv file with your favorite spreadsheet editor:

**
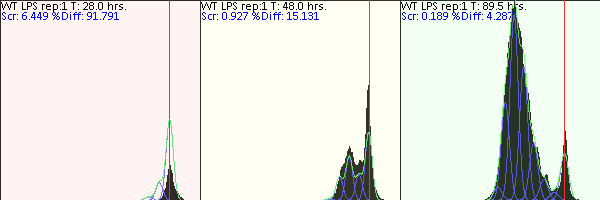

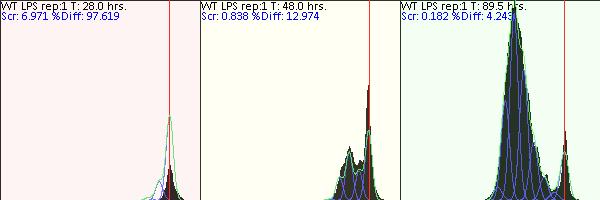
**

Solution Cluster 1 ->

Solution Cluster 2 ->


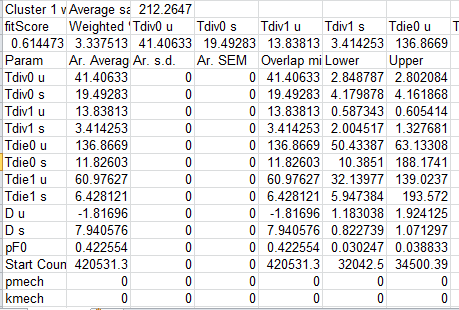


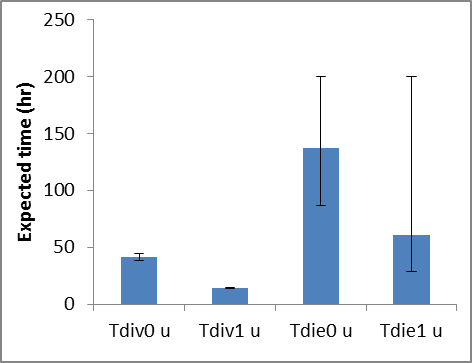

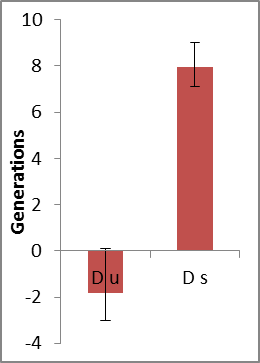

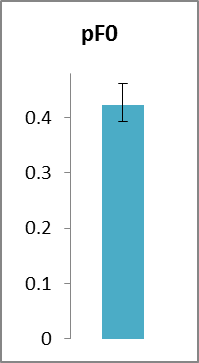
Best fit solutions cluster ranges for each parameter using bar plots can be visualized:

**General Troubleshooting Tips and Suggestions**

Please make sure that the undivided peak is specified accurately for each experimental histogram, as this is used to calculate the positions of all peaks, and can cause large errors during fitting if the wrong peak is selected. A shift parameter is optimized during cell fluorescence fitting to account for small errors in peak selection.

The default number of generations is eight. You can change this if you feel that the data does not include eight peaks or if you feel that more than eight peaks are present.

Try to plan your experiments such that time points are taken before the first division, after the first division but before the second, after a few division have taken place, and after the cells have largely stopped dividing and started dying.

If results are inconsistent when fitting the same dataset multiple times, it is possible that more candidate solutions are required to find the optimal solution. Try increasing the number of candidate solutions until the same solution(s) is discovered consistently.

If you are confident about the time to first division, or the starting cell count (after the initial die-off period caused by experimental manipulation), you can constrain the range of parameters tested using the Advanced Options Pane. Chosen parameter ranges should be reported along with the best-fit parameter ranges.

In some cases it may be important to compensate for “spillover” between channels (i.e. fluorescence is detected across multiple channels for a single fluorophore). FlowMax allows for software compensation of spillover by clicking on the rainbow colored circle to the right of each sample. You can compensate between channels by subtracting a percentage of fluorescence from one channel from the recorded fluorescence value of another channel.

When presenting best-fit parameters, keep in mind that there are two parameters per log-normal distribution reported (e.g. E[Tdiv_0_], s.d.[Tdiv_0_]). Together these parameters, describe the entire distribution. We recommend comparing between populations of distribution curves instead of between pairs of parameters, since the standard deviation parameter can dramatically skew the probability distribution.

If you are having trouble loading a previously saved workspace, this may be a consequence of missing or misplaced FCS files. Please make sure that the relative location of the files does not change with respect to the workspace file. As a last resort, you can edit the workspace files manually in a text editor.

Not all FCS tags are imported into FlowMax such as gating, spillover, time or instrument-specific tags. The exception includes the VOL tag generated by Accuri C6 flow cytometers, which specify the amount of sample volume run. This tag is used to automatically calculate the cell count for each run, allowing for multiple sample sizes to be analyzed accurately. The cell count can be set manually for each run if this information is not available. While it is best to keep the concentrations/experimental conditions consistent throughout the time course to minimize possible bias/errors, you can change the expected number of cells directly (e.g. if you know that only half the volume was used, or half the typical concentration, you can multiply the number of cells directly.

**References**

1. S K, D GC, P VM (1983) Optimization by Simulated Annealing. Science New Series: 671-680.
